# Variability and reliability of effective connectivity within the core default mode network: A multi-site longitudinal spectral DCM study

**DOI:** 10.1016/j.neuroimage.2018.08.053

**Published:** 2018-12

**Authors:** Hannes Almgren, Frederik Van de Steen, Simone Kühn, Adeel Razi, Karl Friston, Daniele Marinazzo

**Affiliations:** a Department of Data Analysis, Faculty of Psychology and Educational Sciences, Ghent University, Belgium; b Center for Lifespan Psychology, Max Planck Institute for Human Development, Berlin, Germany; c Clinic and Polyclinic for Psychiatry and Psychotherapy, University Clinic Hamburg-Eppendorf, Germany; d Monash Institute of Cognitive and Clinical Neurosciences and Monash Biomedical Imaging, Monash University, Clayton, Australia; e The Wellcome Trust Centre for Neuroimaging, University College London, Queen Square, London, WC1N 3BG, UK; f Department of Electronic Engineering, NED University of Engineering and Technology, Karachi, Pakistan

**Keywords:** Dynamic causal modelling, Resting state, fMRI, Effective connectivity, Reliability, Variability, Longitudinal designs

## Abstract

Dynamic causal modelling (DCM) for resting state fMRI – namely spectral DCM – is a recently developed and widely adopted method for inferring effective connectivity in intrinsic brain networks. Most applications of spectral DCM have focused on group-averaged connectivity within large-scale intrinsic brain networks; however, the consistency of subject- and session-specific estimates of effective connectivity has not been evaluated. Establishing reliability (within subjects) is crucial for its clinical use; e.g., as a neurophysiological phenotype of disease progression. Effective connectivity during rest is likely to vary due to changes in cognitive, and physiological states. Quantifying these variations may help understand functional brain architectures – and inform clinical applications. In the present study, we investigated the consistency of effective connectivity within and between subjects, as well as potential sources of variability (e.g., hemispheric asymmetry). We also addressed the effects on consistency of standard data processing procedures. DCM analyses were applied to four longitudinal resting state fMRI datasets. Our sample comprised 17 subjects with 589 resting state fMRI sessions in total. These data allowed us to quantify the robustness of connectivity estimates for each subject, and to generalise our conclusions beyond specific data features. We found that subjects showed systematic and reliable patterns of hemispheric asymmetry. When asymmetry was taken into account, subjects showed very similar connectivity patterns. We also found that various processing procedures (e.g. global signal regression and ROI size) had little effect on inference and the reliability of connectivity estimates for the majority of subjects. Finally, Bayesian model reduction significantly increased the consistency of connectivity patterns.

## Highlights

• Across datasets, subjects’ effective connectivity patterns in the core default mode network showed hemispheric asymmetry.
• Differences in hemispheric asymmetry was found to be a major source of between-subject variability.
• In contrast, most subjects showed reliable within-subject hemispheric asymmetry.
• Differences in preprocessing methods had little effect on connectivity estimates.
• Bayesian model reduction increased the within- and between-subject consistency of connectivity patterns.

## 1 Introduction

During quiet wakefulness the brain shows several patterns of coherent activity, referred to as resting state networks (RSNs; Damoiseaux et al., 2006). RSNs include regions that are both functionally and structurally related (Van Den Heuvel et al., 2009). Most studies characterising resting state networks are based on functional connectivity, which is defined as the statistical dependency among brain signals. However, interactions between brain regions are directed and are therefore not fully captured by (undirected) functional connectivity (Friston, 2011; Razi and Friston, 2016). Studying directed influences between brain regions allows one to assess the relative strength (of reciprocal connectivity) that regions exert on each other, which is thought to be relevant for diagnosis, prognosis, and assessment of treatment responses of neurological and psychiatric disorders (Stephan et al., 2017). Various methods have been developed to infer directed influences between brain regions, among which a prominent framework is Dynamic Causal Modelling (DCM; Friston et al., 2003).

DCM uses Bayesian model inversion procedures to estimate effective connectivity among neural populations from observed signals (e.g., BOLD-signals). It incorporates a biophysically plausible hemodynamic model (i.e., the Balloon model; Buxton et al., 1998) to generate predicted BOLD-responses from neuronal states. Initially DCM was developed to estimate effective connectivity for experimental (task) fMRI studies (Friston et al., 2003). Recently, a particular DCM – referred to as spectral DCM (spDCM) – has been developed to infer effective connectivity in resting state fMRI (Friston et al., 2014). This DCM is based on a generative model of (complex) cross spectra between regional BOLD signals, and uses a power-law function (in the spectral domain) to model (random and endogenous) neuronal fluctuations. Fitting spectral (second-order) data features makes spDCM deterministic, which renders the estimation scheme computationally and statistically more efficient than its stochastic counterpart – that fits the (first-order) timeseries *per se* (i.e., stochastic DCM; Li et al., 2011).

The construct validity of spectral DCM has been established using both simulated and empirical data (Friston et al., 2014; Razi et al., 2015). Friston et al. (2014) simulated resting state fMRI timeseries for a network with three regions. Their results showed that spDCM estimates extrinsic effective connectivity with high accuracy, but tends to underestimate intrinsic connectivity (i.e., the inhibitory influence regions exert on themselves). In a subsequent *in silico* validation study, (Razi et al., 2015) demonstrated a similar accuracy for a network consisting of four regions. Interestingly, both studies showed that the root mean squared error (between the true and estimated connectivity) decreases with the number of scans. Both studies also showed that spDCM is sensitive for detecting group differences in effective connectivity. Most research using empirical data has focused on effective connectivity within the default mode network (e.g., Razi et al., 2015; Sharaev et al., 2016; Ushakov et al., 2016; Zhou et al., 2018). Both Razi et al. (2015) and Sharaev et al. (2016) estimated connectivity within the ‘core’ DMN, which included left and right intraparietal cortices (IPC), medial prefrontal cortex (mPFC), and posterior cingulate cortex (PCC). Both studies found reciprocal positive connectivity between IPC, and positive projections from lateral to medial brain regions. Ushakov et al. (2016) showed that adding extra regions (i.e., left and right parahippocampal gyri) to the four-region DMN did not have a substantial impact on its effective connectivity pattern. Zhou et al. (2018) showed that the salience and dorsal attention network have a negative influence on the core DMN, while the converse influence was slightly positive. Moreover, within the core DMN the same pattern of connectivity was found as in other spectral DCM studies.

In summary, studies that have applied spectral DCM to the DMN have yielded quite consistent results. However, these studies generally focus on group-averaged connectivity. While group studies are very useful to establish predictive validity, a thorough examination of subject and session-specific differences in effective connectivity during rest is an outstanding challenge. Quantifying within-subject stability is especially important in the context of single-patient diagnostics and predictions (Stephan et al., 2017). For other DCMs (e.g., DCM for task fMRI) test-retest reliability has been assessed between a few sessions, and was found to be good to excellent (e.g., Frässle et al., 2015; Schuyler et al., 2010).

Here, we wanted to assess within-subject reliability (and between-subject consistency) of effective connectivity estimated by *spectral* DCM across many resting state fMRI sessions acquired in longitudinal studies. Although effective connectivity during resting state fMRI should be sufficiently reliable to be used in a clinical context, it is likely to vary as a consequence of changes in physical, emotional and cognitive states (e.g., amount of sleep), and the sources of this variability need to be established. Assessment of these longitudinal variations in effective connectivity could yield important insights in the effects of behavioural and psychological states on macroscopic brain dynamics (see, e.g., Laumann et al., 2015). The goals of the present study were to assess whether, and to what extent, connectivity patterns in the default mode network are consistent both within and between subjects, and to investigate the sources of variability in effective connectivity. To meet these aims, we used four longitudinal datasets, with a minimum of ten resting state sessions for each subject. These datasets allowed us to quantify the stability of the posterior estimates of (effective) connectivity in the default mode network across sessions, and to generalise conclusions beyond specific datasets (e.g., subjects' characteristics, scanning parameters, *etc*).

## 2 Methods

### 2.1 Datasets and subjects

Data were obtained from four extensive longitudinal datasets acquired at different research institutions. The total sample consisted of 20 subjects (11 females, age range at start of studies: 24–45 years) with a minimum of 10 resting state sessions for each subject. Altogether, the datasets contained 653 rsfMRI sessions. A summary of the datasets is shown in Table 1.

**Table 1:** Subject information.

Subject	Dataset	M/F	Age^a^	Total sessions^b^	Span^c^	Total scans (time)^d^
S1	MyConn	M	45	89	±1.5 years	518 (±10 min)
S2	Kirby	M	40	156	±3.5 years	200 (±7 min)
S3	Day2day	F	24	50	±5.5 months	150 (±5 min)
S4	Day2day	F	28	13	±3.5 months	150 (±5 min)
S5	Day2day	F	31	50	±13 months	150 (±5 min)
S6	Day2day	M	32	11	±2 months	150 (±5 min)
S7	Day2day	F	29	45	±7 months	150 (±5 min)
S8	Day2day	F	24	47	±5.5 months	150 (±5 min)
S9	Day2day	M	30	43	±7 months	150 (±5 min)
S10	Day2day	F	29	49	±7.5 months	150 (±5 min)
S11	MSC	M	34	10	17 days	818 (±30 min)
S12	MSC	M	34	10	10 days	818 (±30 min)
S13	MSC	F	29	10	12 days	818 (±30 min)
S14	MSC	F	28	10	15 days	818 (±30 min)
S15	MSC	M	27	10	14 days	818 (±30 min)
S16	MSC	F	24	10	15 days	818 (±30 min)
S17	MSC	F	31	10	39 days	818 (±30 min)
S18	MSC	F	27	10	18 days	818 (±30 min)
S19	MSC	M	26	10	16 days	818 (±30 min)
S20	MSC	M	31	10	21 days	818 (±30 min)

Abbreviations: MyConn = Myconnectome, MSC = Midnight Scan Club.

a Age at the start of study.

b Total number of sessions initially included in the present study (these differ slightly from the original studies because some low-quality images were not shared, duplicates were encountered, or initial pilot sessions were not included).

c Span = approximate time over which all rsfMRI scan sessions took place.

d Total number of scans in each session

*Dataset 1.* The first dataset (‘myconnectome’) was part of the MyConnectome project (see, Laumann et al., 2015). During this project, resting state fMRI scans were acquired from a single person (male, 45 years at the start of study) on 89 occasions over the period of 1.5 years. MRI data were obtained with a Siemens MAGNETOM Skyra 3T MRI scanner (Siemens, Erlangen, Germany), using a 32-channel head coil. Resting state fMRI was acquired using a multi-band echo-planar imaging (MBEPI) sequence (TR = 1160 ms; TE = 30 ms; voxel size = 2.4 mm × 2.4 mm x 2 mm; FOV = 230 mm; flip angle = 63°; multi-band factor = 4). Functional images for the first 14 sessions contained 68 slices; images from remaining sessions comprised 64 slices. Resting state scan length was approximately 10 min (518 images). T1 images were acquired using a MPRAGE sequence (TR = 2400 ms; TE = 2.14 ms; TI = 1000 ms; voxel size = 0.8 mm isotropic; 256 sagittal slices; flip angle = 8°; GRAPPA factor = 2). Only the T1-weighted image acquired during the session prior to the first rsfMRI session was used in the present study (i.e., for coregistration and normalization). This dataset was obtained from the OpenfMRI database. Its accession number is ds000031.

*Dataset 2.* The second dataset (‘Kirby’) contained data acquired from a single subject (40 years at the start of study, male) on 156 occasions (3.5 years; see, Choe et al., 2015). The subject was scanned using a 3T Philips Achieva scanner (Philips Healthcare, Best, Netherlands), with a 16-channel neurovascular coil. Functional resting state data were acquired using a multi-slice SENSE-EPI sequence (TR = 2000 ms; TE = 30 ms; voxel size = 3 mm × 3 mm x 3 mm; flip angle = 75°; 37 axial slices; SENSE factor = 2). Scan length was approximately 7 min (200 images). T1-weighted images were acquired using a MPRAGE sequence (TR = 6.7 ms; TE = 3.1 ms; TI = 842 ms; voxel size = 1.0 mm × 1.0 mm × 1.2 mm; flip angle = 8°; SENSE factor = 2). The T1-weighted image acquired during the first scan session was used in the present study.

*Dataset 3.* The third dataset (‘day2day’) contained data acquired from eight subjects (6 females; age range 24–32 years; Filevich et al., 2017). The number of scan sessions per subject ranged from 11 to 50 (sessions in total), and were acquired within a period of 2–13 months. Subjects were scanned using a 3T Magnetom Trio MRI scanner (Siemens, Erlangen, Germany) and a 12-channel head coil. RsfMRI data was acquired using a T2*-weighted echo planar imaging (EPI) sequence (TR = 2000 ms; TE = 30 ms; voxel size = 3 mm × 3 mm × 3 mm; flip angle = 80°; 36 axial slices; GRAPPA acceleration factor = 2). The length of resting state scanning was approximately five minutes (150 images). Structural MRI scans were acquired using a MPRAGE sequence (TR = 2500 ms; TE = 4.77 ms; TI = 1100 ms; voxel size = 1.0 mm × 1.0 mm × 1.0 m; flip angle = 7°). Only the T1-weighted image acquired during the first scan session was used.

*Dataset 4.* The fourth dataset (‘midnight scan club’) contained data from ten subjects (5 females; age range 24–35 years; see, Gordon et al., 2017). Participants were scanned at midnight on twelve consecutive days with a 3T Siemens Trio MRI scanner (Siemens, Erlangen, Germany). On ten occasions, rsfMRI data were acquired with a gradient-echo EPI sequence (TR = 2200 ms; TE = 27 ms; voxel size = 4 mm × 4 mm × 4 mm; flip angle = 90°; 36 axial slices). Each session contained 818 vol (approximately 30 min). Structural scans were acquired using a gradient-recalled inverse recovery (GR-IR) sequence (TR = 2400 ms; TE = 3.74 ms; TI = 1000 ms; voxel size = 0.8 mm × 0.8 mm × 0.8 mm; 224 sagittal slices; flip angle = 8°). The first structural image acquired from each subject was used for the analyses. This data was obtained from the OpenfMRI database. Its accession number is ds000224.

### 2.2 Data analyses

#### 2.2.1 Preprocessing

Preprocessing was performed using the SPM12 software package (revision 6906; Wellcome Centre for Human Neuroimaging; www.fil.ion.ucl.ac.uk/spm/software/spm12). The first five images of each session's rsfMRI sequence were discarded to allow for T1 equilibration. First, resting state fMRI images were corrected for differences in slice timing (using the central slice of each volume as a reference). Next, images were realigned to the first functional volume of each session. Images were then coregistered to the skull-stripped anatomical image. Finally, images were normalized to MNI space (Montreal Neurological Institute) and smoothed using a Gaussian kernel (6 mm FWHM).

#### 2.2.2 Time-series extraction

**Fig. 1:**
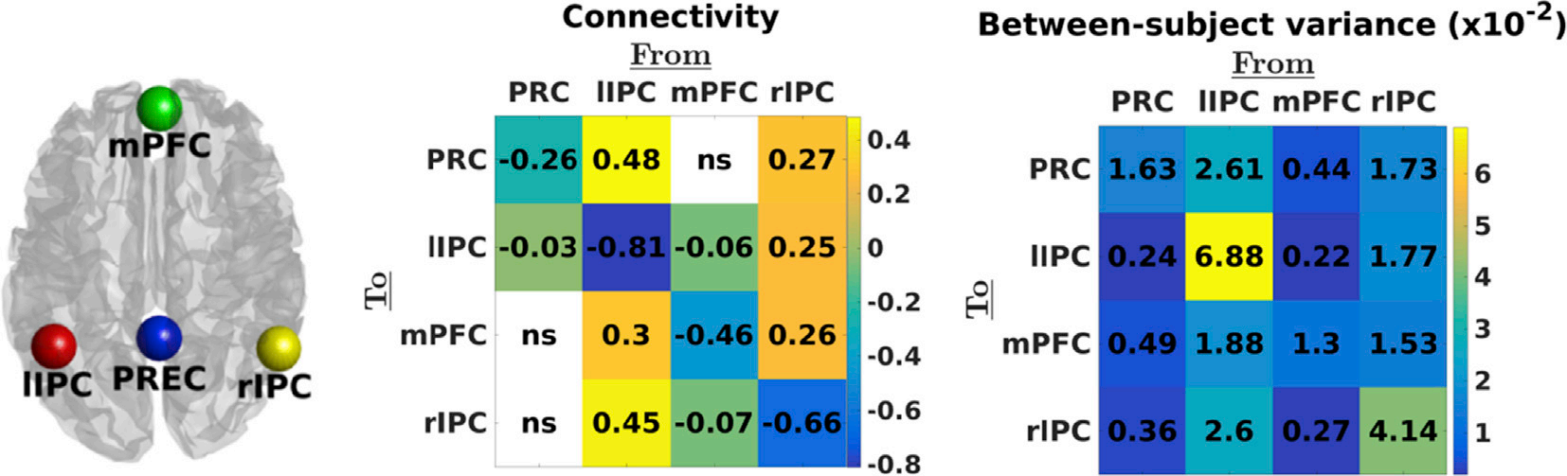
Left panel: location of ROIs used in the present study. Middle panel: Estimated effective connectivity (from columns to rows) at the group level. Diagonal elements reflect self-inhibition parameterised in log-scale (relative to the prior mean of −0.5 Hz). A posterior probability criterion of 90% was used (*ns* depicts not significant, i.e., posterior probability below 90%). Right panel: estimated between-subject variability for each connection (PEB.Ce). It is evident that the left and right IPC showed the greatest between-subject variability in self-inhibition (log-scale) and extrinsic connectivity (hertz).

Session-specific DMN voxels were identified by specifying and estimating a GLM containing: (1) a discrete cosine basis set as principal regressor (frequency range: 0.0078–0.1 Hz), (2) six head motion regressors (three translational, three rotational), (3) a regressor for CSF signal (principal eigenvariate of 5 mm ROI within CSF circulation system), and (4) a regressor containing WM signal (principal eigenvariate of 7 mm ROI within brainstem). The number of cosine components depended on the number of scans within a session. An F-contrast was specified across all DCT components to produce an SPM (testing for high amplitude fluctuations in the frequency range model), which was masked using ROIs (sphere radius = 10 mm) extracted from template ICA maps (Smith et al., 2009). ROI centre coordinates were (x = 2; y = −58; z = 30) for precuneus, (x = 2; y = 56; z = −4) for medial prefrontal cortex, (x = −44; y = −60; z = 24) for the left inferior parietal cortex, and (x = 54; y = −62; z = 28) for the right inferior parietal cortex (see, Fig. 1: left panel). Coordinates were labelled using the AAL atlas. Time-series were acquired by computing the principal eigenvariate of signals from voxels centred on the peak voxel of the aforementioned F-contrast (session-specific; sphere radius = 8 mm) within each ROI. This procedure accommodated session- and subject-specific differences in the exact location of DMN regions. Voxels were only included if they survived an a priori specified threshold: if sessions contained less than 200 scans per session, voxels were included if they exceeded an uncorrected (full brain) alpha-threshold of 0.05. If sessions contained more scans, a stepwise increase in alpha-threshold (alpha = 0.001, 0.01, to 0.05) was applied, until significant voxels were detected (using an upper boundary of alpha = 0.05). Importantly, the alpha-level specified here is used to detect voxels that contain low-frequency fluctuations, and is independent from the criterion used to infer connectivity.

#### 2.2.3 (Spectral) Dynamic causal modelling and hierarchical Bayes

Spectral DCM for resting state fMRI (Friston et al., 2014) estimates the intrinsic effective connectivity (i.e., the ‘A-matrix’) between brain regions from observed BOLD responses, taking into account (modelled) effects of (neuro)vascular processes and (spectral) noise at different levels. DCM models the *rate of change* in activity (per second) of one region as a function of activity in another region, therefore expressing effective connectivity in Hertz (Hz). Since DCM uses Bayesian estimates of effective connectivity, each parameter (i.e., connection strength) is equipped with a prior density. The priors for extrinsic effective connections in DCM are shrinkage priors, which are minimally informative with respect to the sign of the connection but preclude extreme values. Intrinsic (self) connections have more informative priors to reflect cortical gain control and the ensuing stability of neuronal dynamics. To estimate parameters at the group-level, a hierarchical or Parametric Empirical Bayesian framework (PEB; Friston et al., 2016) is used to model effects at multiple levels (e.g. session- and subject-level). At all levels, parameters (e.g., between-subject variance) are supplemented with appropriate priors (in fixed proportion to the above priors on within-subject connectivity). Precision components specify whether the variance of between-subject (and between-session) random effects is equal or differs for connectivity parameters.

In the present study, DCMs (with all possible connections among regions) were specified and inverted for each session separately (DCM12; revision 6801), without the specification of exogenous (i.e., experimental) inputs. Default priors implemented in SPM were used for all parameters at this level. Sessions were excluded from analysis if they did not meet five diagnostic criteria; namely, (1) explained variance of predicted BOLD signals above 60%, (2) at least one connection with a connection strength greater than 1/8 Hz, (3) at least one effectively estimated parameter (based on Kullback-Leibler divergence of posterior from prior distribution), (4) maximum frame-wise displacement (FD) under 1.5 mm, and (5) a maximum alpha-threshold of 0.05 for which significant voxels were found in all DMN regions for that session. Three subjects (i.e., S13, S18, and S19) were rejected because they had less than 8 sessions after rejection based on diagnostic checks. Additionally, thirty-one sessions (across subjects and datasets) were excluded because they did not meet diagnostic criteria, and three sessions were discarded because of other problems (e.g., incomplete volumes). A total of 589 rsfMRI sessions were included after quality and diagnostic checks.

To compute average connectivity at the subject-level we specified Parametric Empirical Bayes (PEB; Friston et al., 2016) models with one regressor (comprising a column of ones) that modelled average (within-subject) connectivity over sessions. The subject-specific PEB models were then included in a group-level PEB model, including again one regressor to model average (between-subject) connectivity over subjects. Default settings were used for estimation at the subject and group level; i.e., the prior covariance of connections had the same form at the session, subject and group-level, where the between-session and between-subject prior covariance was 1/16th of the prior covariance of connections at the session-level (see, Friston et al., 2016). Each parameter was equipped with a separate between-subject precision component. Only effective connectivity parameters were included as dependent variables in subject and group analyses. Inference concerning effective connectivity (at all levels) was based on a posterior probability criterion of 90% for each connection.

#### 2.2.4 Stability criteria

Stability of the strength and direction of connections was assessed for three different network characteristics. (1) We tested hemispheric asymmetry for each session and subject by computing the posterior probability that the average outgoing connectivity (i.e., efferent or out-degree) from the left IPC differed from the average outgoing connectivity from the right IPC (posterior probability criterion = 90%). The ‘asymmetry index’ was computed as the average outgoing connectivity from left minus the average outgoing connectivity from right IPC. Between (resp., within) subject stability of asymmetry was evaluated by computing the ratio of subjects (resp., sessions) that showed most prominent influence from either right or left IPC. (2) We assessed stability of the estimated connectivity matrices (i.e., over all connections) by calculating the average correlation between vectorised connectivity matrices for each pair of subjects and sessions. (3) We assessed between (resp., within) subject stability of the type of connection (i.e., excitatory, inhibitory, or no influence) by computing the percentage of subjects (resp., sessions) that showed either a positive, negative, or non-existent influence between regions (using the 90% posterior confidence criterion). The latter stability measure was computed for each connection separately.

#### 2.2.5 Effect of (pre)processing steps

The effect of three (pre)processing steps on connectivity and stability was assessed. (1) (Empirical) Bayesian model reduction was applied to assess the effect of using subject-specific and group connectivity as empirical priors for session- and subject-specific estimation, respectively. We compared stability with and without empirically optimising connectivity parameters at the session and subject level (as implemented by spm_dcm_peb.m). (2) To assess the effect of ROI size on connectivity and stability, we reanalyzed the data using spherical ROIs with radii of 4 mm, 8 mm, 12 mm and 16 mm. ROIs were centred at the average coordinate of the (session-specific) voxels included in the previous ‘basic’ analyses. To allow proper comparison, we calculated the eigenvariate of all significant voxels in the sphere (i.e., without using the conjunction with the ROI derived from the ICA template). The same subjects were excluded as in the basic analyses. To ensure proper comparison between analyses with different ROI sizes, sessions were excluded if they did not reach diagnostic thresholds for all ROI sizes. Consequently, 57 sessions were discarded, which yielded a total sample of 563 sessions. (3) Finally, we assessed the influence of global signal regression (GSR) on the reliability and connectivity. Therefore we repeated the ‘basic’ analyses with GSR, which was done by scaling (preprocessed) fMRI volumes with the inverse of the scan-specific global mean intensity (in SPM: global normalization = ‘scaling’). All subsequent analyses (e.g., peak-value coordinate detection, time-series extraction) were performed using these scaled images. Again, same subjects were excluded as in the basic analyses. Forty-two sessions were excluded because they did not reach diagnostic thresholds for one or both analyses (i.e., with or without GSR), which left a sample of 578 rsfMRI sessions.

#### 2.2.6 Statistical inference

At each level of the hierarchy (i.e., session, subject, and group) a posterior density was estimated for each connection (constituting a multivariate distribution over parameters). Inference concerning connection strengths (see, e.g., Fig. 1) was based on these posterior densities. Inference regarding asymmetry was based on a contrast (i.e., difference) of connections arising from left and right IPC. The resulting density was then used to evaluate the significance of asymmetry. For inference regarding other metrics (e.g., regarding the within-subject reliability of asymmetry, or the effect of processing steps), statistical tests were performed using maximum-a-posteriori (MAP) estimates as summary statistics (e.g., representing connection strength or asymmetry). For each specific context, an appropriate test was performed (e.g., standard binomial test for reliability of asymmetry, randomization testing for the effect of preprocessing on reliability) to establish classical statistical significance.

## 3 Results

### 3.1 Group-level

Estimated connectivity at the group level is shown in Fig. 1. Connections from bilateral IPC were stronger compared to other connections. Moreover, the left IPC showed a stronger outgoing influence compared to right IPC (mean difference = 0.15; SD = 0.03; posterior probability > 0.99) and the lowest self-inhibition. Between-subject variability for both extrinsic (i.e., between-regions) and intrinsic connections (i.e., self-inhibition) was greater for projections arising from left and right IPC compared to other regions.

### 3.2 Between-subject variability

#### 3.2.1 Between-subject variability in hemispheric asymmetry

**Fig. 2:**
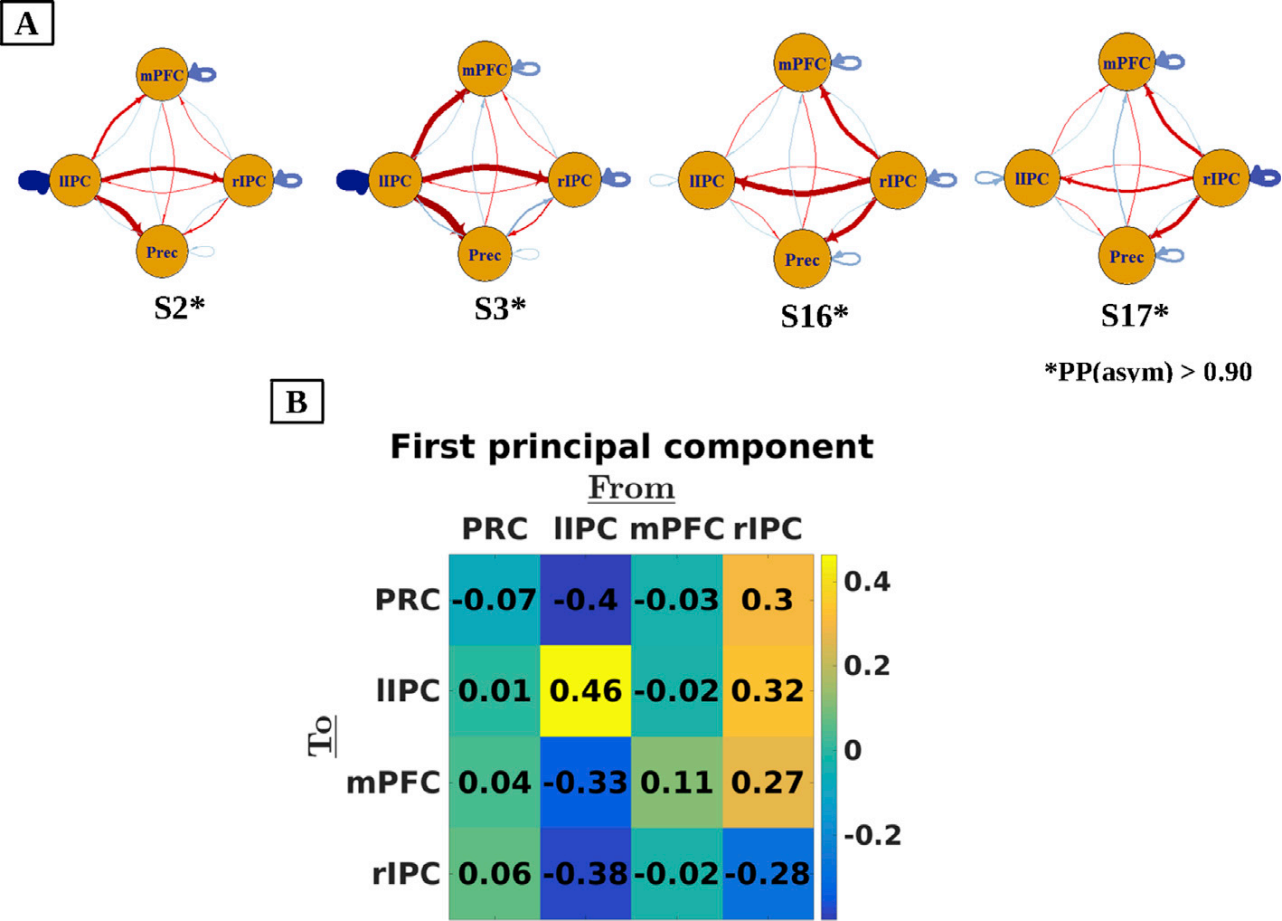
Panel A: Connectivity patterns for exemplar subjects. For extrinsic connections (i.e., between regions), red lines denote positive connectivity and blue lines negative connectivity. For self-connections, red lines depict connectivity above the prior mean, while blue lines depict connectivity below the prior mean (i.e., −0.5 Hz). Line thickness and brightness reflect the strength of the respective connection. Across datasets, subjects showed most dominant influence from either left (e.g., S2 and S3) or right IPC (e.g., S16 and S17). Moreover, self-inhibition was lowest for the dominant IPC in 15 subjects. Panel B: Loadings on the first principal component of effective connectivity across subjects. Coefficients of the left and right IPC show opposite signs. Self-connections (shown on diagonal in negative log-scale) have opposite loadings compared to ipsilateral extrinsic (off-diagonal) connections, which indicates that subjects with high extrinsic influence show high self-inhibition of the ipsilateral IPC.

Average connectivity for some exemplar subjects is shown in Fig. 2 (panel A; see, Fig. S1 for connectivity estimates of all subjects). All but one subject showed a dominant influence from either left or right IPC. Post-hoc tests comparing average outgoing connectivity from left IPC to average connectivity from right IPC indeed confirmed our observations: ten subjects showed significantly higher influence from left IPC, while six subjects showed significantly greater influence from right IPC (PP > 0.90). The average difference between left and right IPC connectivity was 0.60 Hz, indicating a non-trivial effect (about six times the heuristic threshold of 0.1 Hz used in DCM studies; see, e.g., Razi et al., 2015). The dominant IPC showed lowest self-inhibition in fifteen out of sixteen asymmetric subjects. The predominance of asymmetry in between-subject variability was confirmed with a principal component analysis (PCA) on effective connectivity across subjects. The first principal component showed highest (and opposite) loadings on left and right IPC (see, Fig. 2 panel B), and explained approximately 62% of total variance. Self-connections showed opposite loadings compared to ipsilateral extrinsic connections, which suggests that subjects with high extrinsic influence show high self-inhibition of ipsilateral IPCs.

#### 3.2.2 Effective connectivity in terms of (out-degree) dominant versus non-dominant

**Fig. 3:**
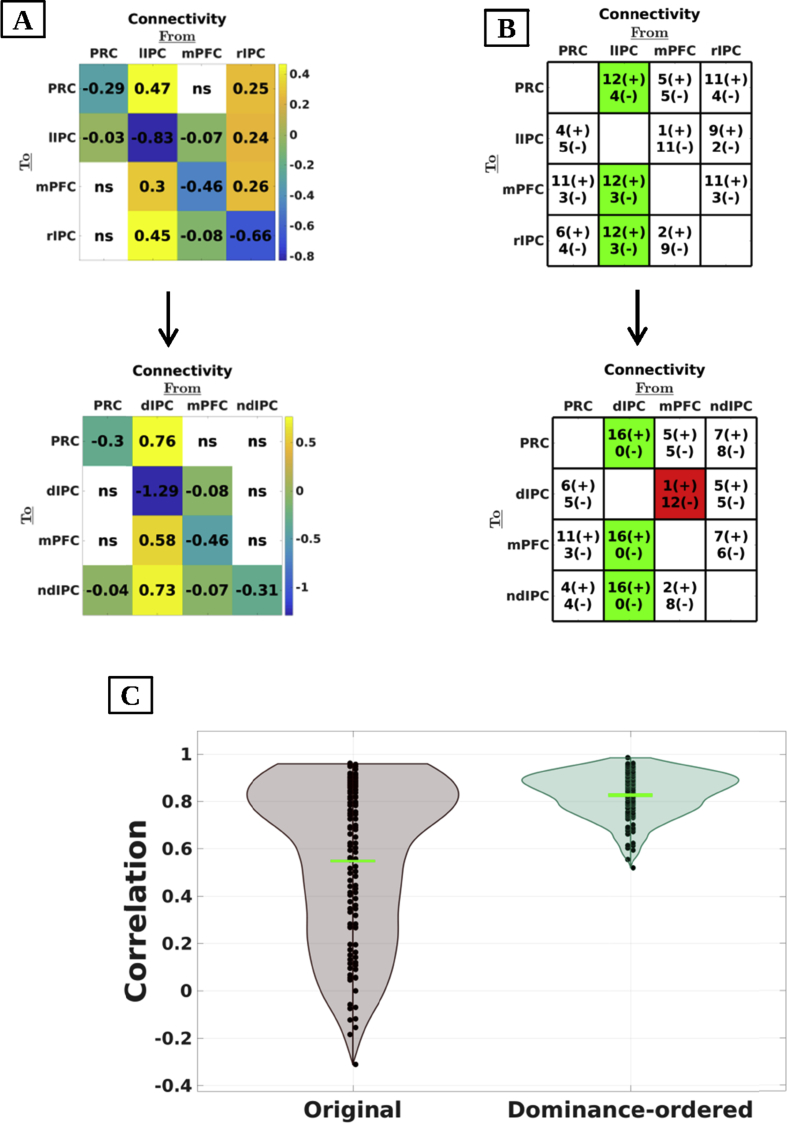
Panel A: Effective connectivity at group-level using original and dominance-ordered matrices. When hemispheric dominance was accommodated at the subject-level, results suggest that the non-dominant hemisphere has no outgoing effective connectivity and is slightly inhibited by medial regions. *Ns* depicts not significant (i.e., posterior probability below 90%). Panel B: Number of subjects with excitatory (positive) or inhibitory (negative) influences (posterior probability > 90%; effective connectivity from column to row regions). Connections that were positive or negative in more than 70% of subjects are shown in green or red, respectively. Self-connections are omitted for simplicity. The dominant IPC showed positive influence on all other regions, while the influence of the non-dominant IPC differed between subjects. Moreover, the left mPFC exerted inhibitory influence on the dominant IPC in 12 out of 16 (75%) asymmetric subjects. Panel C: Violin plots of correlations between left-right (left plot) and dominance-ordered (right plot) connectivity-matrices for all possible pairs of subjects. Horizontal green lines depict the mean correlation. The consistency is significantly greater when hemispheric dominance was taken into account. Anatomical labels: PRC = precuneus; mPFC = medial prefrontal cortex; dIP = dominant inferior parietal cortex; ndIP = non-dominant inferior parietal cortex; l/rIPC = left/right inferior parietal cortex.

Next, we accommodated between-subject differences in asymmetry by rearranging the connectivity matrices at the subject-level (and their respective (co)variances) from a left-right orientation to a dominance-related orientation. Specifically, we swapped left and right IPC for subjects that showed significant right asymmetry. This produced reordered connectivity matrices for which the second column and row represented the dominant hemisphere and the fourth column and row represented the non-dominant IPC in all subjects. The subject with a low evidence for hemispheric asymmetry was excluded from these analyses. First, we assessed the effect of accounting for asymmetry on group-level connectivity estimates. Therefore we re-estimated group-level connectivity (i.e., by re-inverting the second level PEB model) using dominance-ordered connectivity matrices (see, Fig. 3: Panel A). When effective connectivity (at the subject-level) was casted in terms of dominant versus non-dominant IPC, the non-dominant IPC showed no outgoing connectivity at the group-level, and was slightly inhibited by medial regions (PP > 0.90).

Second, we assessed between-subject consistency in terms of connection type using original and re-ordered connectivity matrices (using subject-level matrices). Therefore, we enumerated (for each connection separately) the number of subjects showing excitatory, inhibitory, or non-existent influence (i.e., with a posterior probability < 90%). The results are shown in Fig. 3 (Panel B). After accommodating hemispheric asymmetry, the results showed that the dominant IPC exerted a positive influence on all other regions for all subjects, while the influence of the non-dominant IPC varied between subjects. Moreover, connectivity from the mPFC to dominant IPC was negative in 12 out of 16 subjects (75%).

Third, we assessed the change in similarity of connectivity patterns between subjects after taking into account asymmetry. Therefore, we calculated correlations between (vectorised) connectivity matrices for all possible pairs of asymmetric subjects (120 pairs in total) for both left-right and dominance-ordered connectivity matrices (estimates at subject-level). The results of this analysis are shown in Fig. 3 (Panel C). The ensuing average correlation was 0.55 (range: [-0.31 0.96]) for the left-right and 0.83 (range: [0.52 0.98]) for the dominance-ordered connectivity matrices. To test whether the increase in correlation was significantly higher than what would be expected under the null-hypothesis (i.e., that left-right dominance swapping does not lead to a greater correlation), we performed a randomization-based statistical test. Specifically, we randomized the matrix entries that represented extrinsic connectivity from either left or right IPC. This was done pair-wise, meaning that connectivity to a region was switched with connectivity to that same region but from the contralateral IPC. We swapped the columns and rows of all permuted matrices for which the left IPC showed greater outgoing connectivity compared to the right IPC. We then computed the average correlation between all pairs of subjects. The difference between the correlations – with and without swapping – for all permutations constituted our (empirical) null-distribution. The number of randomizations was set to 5000. Results from the empirical data showed that the increase in between subject similarity was significantly greater than what could be expected under the null-hypothesis (Effect size = 0.28, p < 0.001). Thus, casting effective connectivity in terms of (out-degree) *dominant* versus non-*dominant*, as opposed to *right* versus *left*, hemisphere markedly (and significantly) improved between-subject consistency. In short, interesting and systematic connectivity patterns emerge when between-subject differences in hemispheric asymmetry are taken into account.

### 3.3 Within-subject variability

#### 3.3.1 Within-subject reliability of asymmetry and connection type

**Fig. 4:**
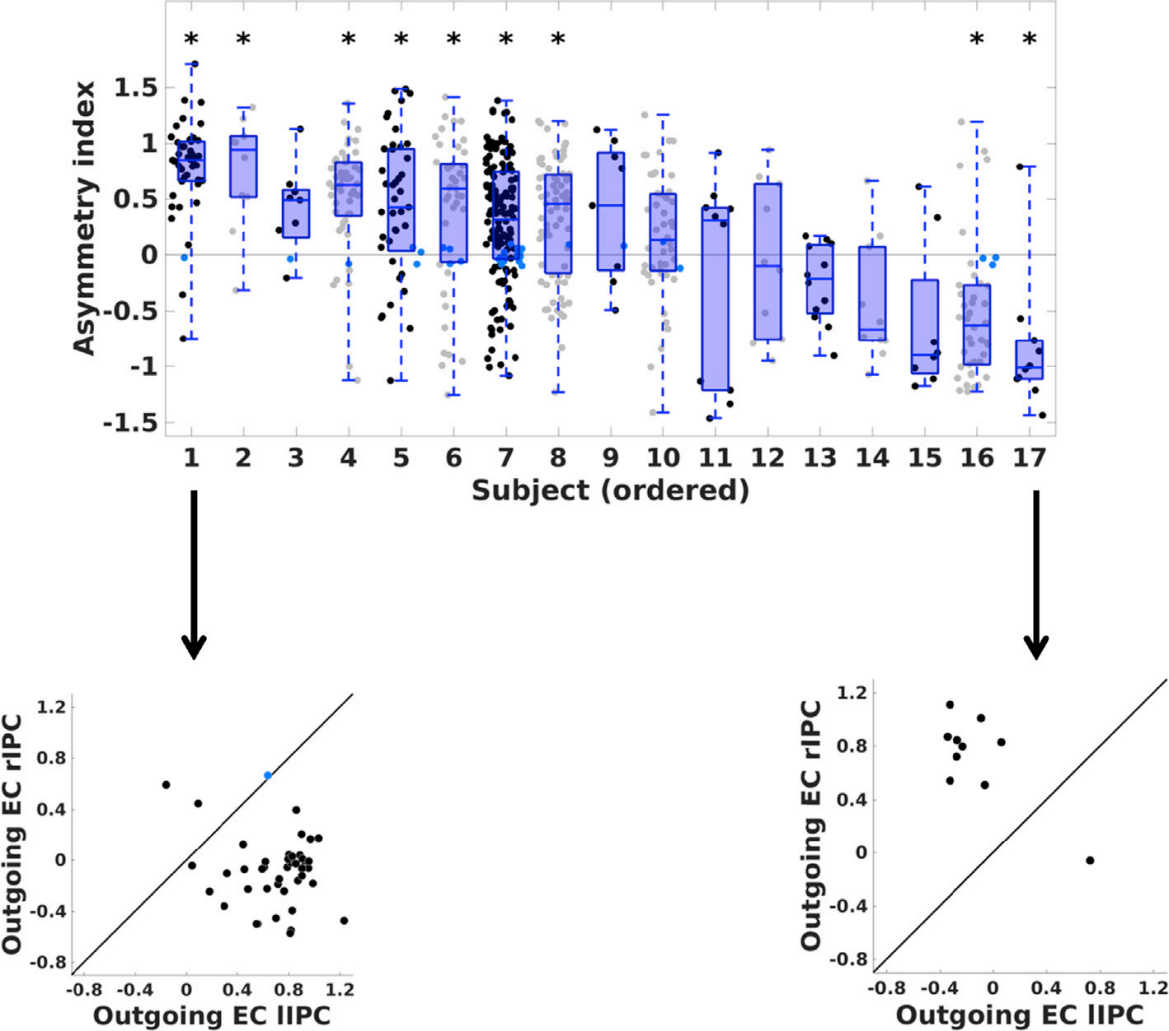
Upper panel: Session-specific hemispheric asymmetry for all subjects, in order of decreasing proportion of left hemisphere-dominant sessions. The asymmetry index was computed as the average efferent influence from the left minus the mean efferent influence from the right IPC. Dots represent the asymmetry index for each session of the respective subject (positive = left dominant; negative = right dominant). Blue dots depict sessions without evidence for hemispheric dominance. Asterisks above data clouds indicate subjects with stability in asymmetry significantly different from zero (FDR = 5%). Blue box plots of subject-specific asymmetry indices are superimposed on data clouds. Lower panels: Hemispheric asymmetry for the most stable left and right asymmetric subject. Black circles represent the average outgoing influence from left (x-axis) and right IPC (y-axis). Circles below the reference line indicate sessions with higher influence from left IPC, circles above the reference line depict sessions with higher influence from right IPC. Light-blue circles depict sessions for which asymmetry did not survive the posterior probability criterion.

The same tests for hemispheric asymmetry (i.e., comparing average connectivity from left versus average connectivity from right IPC) were performed on every session's connectivity matrix. Results for *all* subjects are shown in Fig. 4. In total 94% of sessions showed significant lateralization (PP > 0.90). For subjects showing significant asymmetry at the subject-level (N = 16) on average 71% (range: [0.4 0.93]) of sessions showed the same asymmetry as the respective subject-specific asymmetry. To test which subjects showed significant stability in asymmetry (i.e., left or right dominance), we performed a (two-tailed) binomial test for each subject separately. To correct for multiple comparisons (N = 16; symmetric subject left out) we applied the Benjamini-Hochberg method (Benjamini and Hochberg, 1995) using a false-discovery rate (FDR) of 5%. To allow proper comparison to a chance level of 50%, sessions without significant asymmetry (6%) were discarded from these analyses. Effect sizes showed that twelve asymmetric subjects had at least 70% of sessions with the same (left versus right) asymmetry, statistical analyses revealed that nine subjects had a consistent asymmetry that was significantly different from 50% (see, Table 2).

**Table 2:** Stability of asymmetry in effective connectivity within the DMN. The second column reports the number of sessions (and percentage) that showed significant asymmetry (i.e., irrespective of hemisphere) for each subject. The third column depicts the proportion of asymmetric sessions that showed the same dominance as the respective subject. Effect sizes of at least 70% are shown in bold. The fourth column shows the p-values for the proportion of asymmetric sessions for each subject, not corrected for multiple comparisons. The final column shows the p-values after correction for multiple comparisons using the Benjamini-Hochberg method (FDR = 5%). FDR-corrected p-values lower than 0.05 are shown in bold.

Subject	Number Asym. Sessions (%)	Effect size (%)	Unc p-value	FDR-cor p-value
S1	74 (99%)	**71.6**	0.000	**0.001**
S2	135 (89%)	**74.8**	0.000	**0.000**
S3	48 (98%)	**87.5**	0.000	**0.000**
S4	12 (100%)	66.6	0.388	0.443
S5	46 (96%)	60.9	0.184	0.268
S6	8 (89%)	**87.5**	0.070	0.113
S7	41 (91%)	**75.6**	0.001	**0.003**
S8	43 (93%)	**88.4**	0.000	**0.000**
S9	40 (93%)	**77.5**	0.001	**0.002**
S10	42 (98%)	**95.2**	0.000	**0.000**
S11	10 (100%)	**90.0**	0.021	**0.038**
S12	10 (100%)	**90.0**	0.021	**0.038**
S14	10 (100%)	40.0	0.754	0.754
S16	8 (100%)	**75.0**	0.289	0.385
S17	10 (100%)	**70.0**	0.344	0.423
S20	8 (89%)	62.5	0.727	0.754

**Fig. 5:**
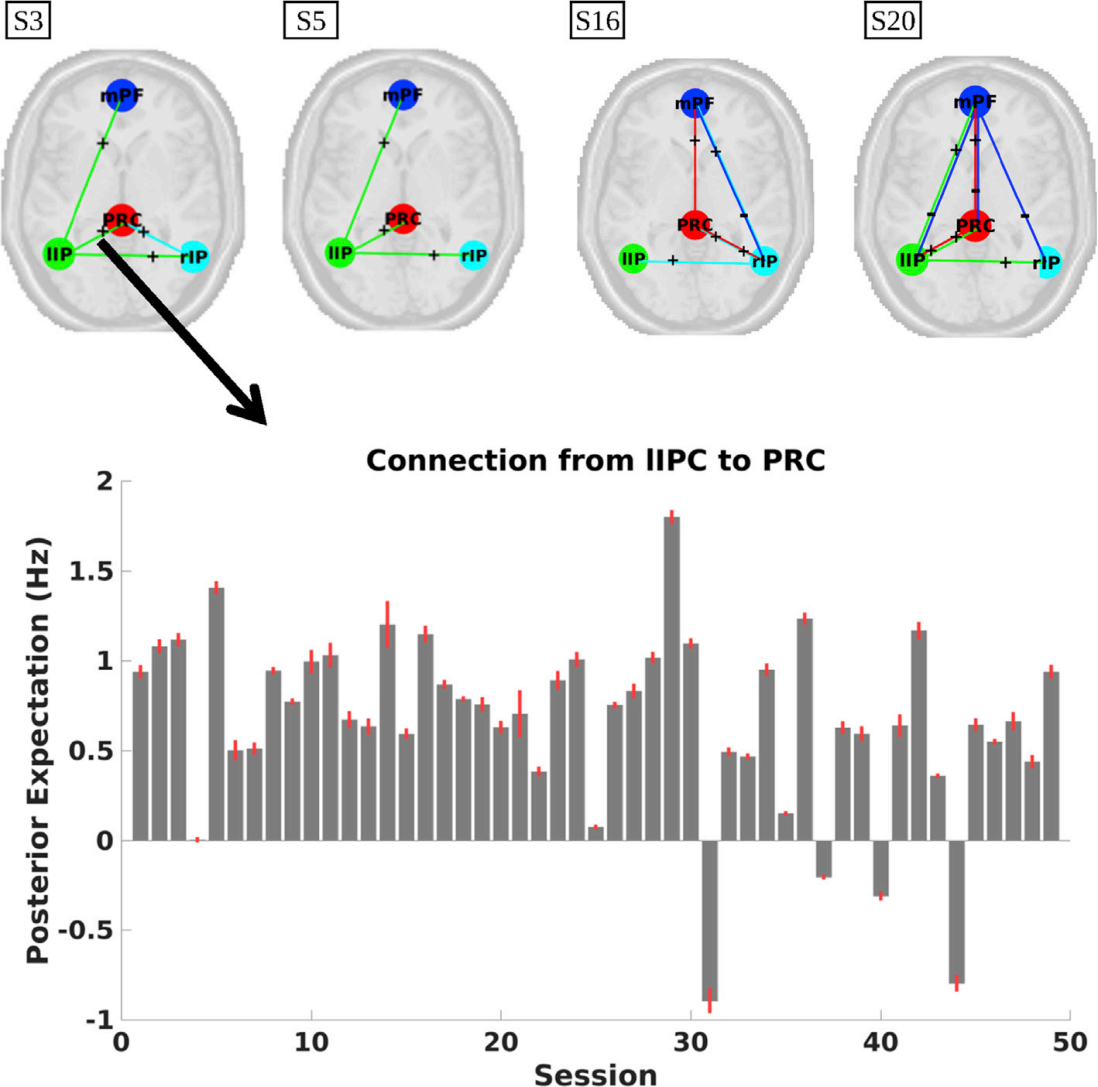
Upper panel: Connections having the same sign in at least 75% of sessions within the respective (exemplar) subject. The line colours depict the source of a connection (e.g., green lines depict connections from the lIPC to other regions). Stable connections arise from left or right IPC, which nicely coincides with the subject-specific asymmetry. For visualization purposes the precuneus is shown more anteriorly than in reality. Lower panel: Posterior estimates of the strongest connection for subject 3, plotted against session number.

In subsequent analyses we assessed the sign-stability of connections (i.e., inhibitory, excitatory, or non-existent) within subjects (including all sessions). Fig. 5 shows connections that had the same influence (i.e., inhibitory or excitatory) in at least 75% of sessions for exemplar subjects (similar figures for all subjects are shown in Supplementary Fig. 2). Subjects showed notably stable positive connectivity from either left or right IPC (e.g., subject 3 and subject 16, respectively), which nicely coincides with the average subject-specific hemispheric asymmetry reviewed in the previous paragraph. Additionally, each subject showed unique stable connections.

#### 3.3.2 Characterization of within-subject variability

To characterize variability in effective connectivity within subjects, we performed principal component analyses on effective connectivity for each subject separately. Subjects for which hemispheric asymmetry was an important explanation of within-subject variability were detected using the following criteria. If two out of three highest (positive) values loaded on outgoing connections from left (right) IPC, while two out of three lowest (i.e., most negative) values loaded on outgoing connections from right (left) IPC, respectively, subjects were considered to have within-subject variability in asymmetry. Results showed that the first principal component of 8 subjects had such opposite loadings on left versus right IPC, accounting on average for 40% (range: 24–73%) of between-session variance (see, Supplementary Table 1). Additionally, for two subjects (S2 and S19) between-session variability was characterized by opposite and high loadings on (outgoing) connectivity from right IPC and precuneus.

#### 3.3.3 Effective connectivity in terms of (out-degree) dominant versus non-dominant

Next, we investigated whether within-subject stability would increase more than expected from chance if session-specific asymmetry is taken into account. We therefore compared the reliability of connectivity patterns before and after re-arranging connectivity matrices from a left-right to a dominance-related order. Reliability was computed as the average correlation across all possible pairs of sessions for both original and reordered matrices. Randomization testing (as described in the previous section) was performed for each subject separately. Results were corrected for multiple comparisons (N = 16) using the method of Benjamini-Hochberg (Benjamini and Hochberg, 1995) with an FDR of 5%. The average increase in correlation after re-arranging matrices was 0.13 (range: 0.04–0.34). Analyses revealed that 12 out of 16 subjects showed significantly increased reliability when asymmetry was taken into account.

### 3.4 Effect of (Pre)processing

#### 3.4.1 Bayesian model reduction (BMR)

At the subject level, hemispheric asymmetry was not flipped (i.e., from left to right or vice versa) in any subject after including empirical priors. The similarity between *subject-specific* connectivity matrices increased after empirical BMR (Effect size = 0.057). To test the statistical significance of this increase, we randomized labels (i.e., before versus after BMR) in a pair-wise way and computed the difference in average correlation for each randomization. These values formed our empirical null-distribution for subsequent inference. The number of randomizations was 50000. Randomization testing showed that the increase in average correlation after BMR was highly significant (Effect size = 0.057; p < 0.001). Correlations between *session-specific* connectivity matrices increased for 14 out of 17 subjects after BMR (average increase in correlation was 15.4%). The same randomization test described above was used, with the Benjamini-Hochberg correction using a false-discovery rate (FDR) of 5% (N = 17). 10000 randomizations were performed for each subject. This procedure showed that the increase in correlation was significant for 7 subjects.

#### 3.4.2 Effect of ROI size

**Fig. 6:**
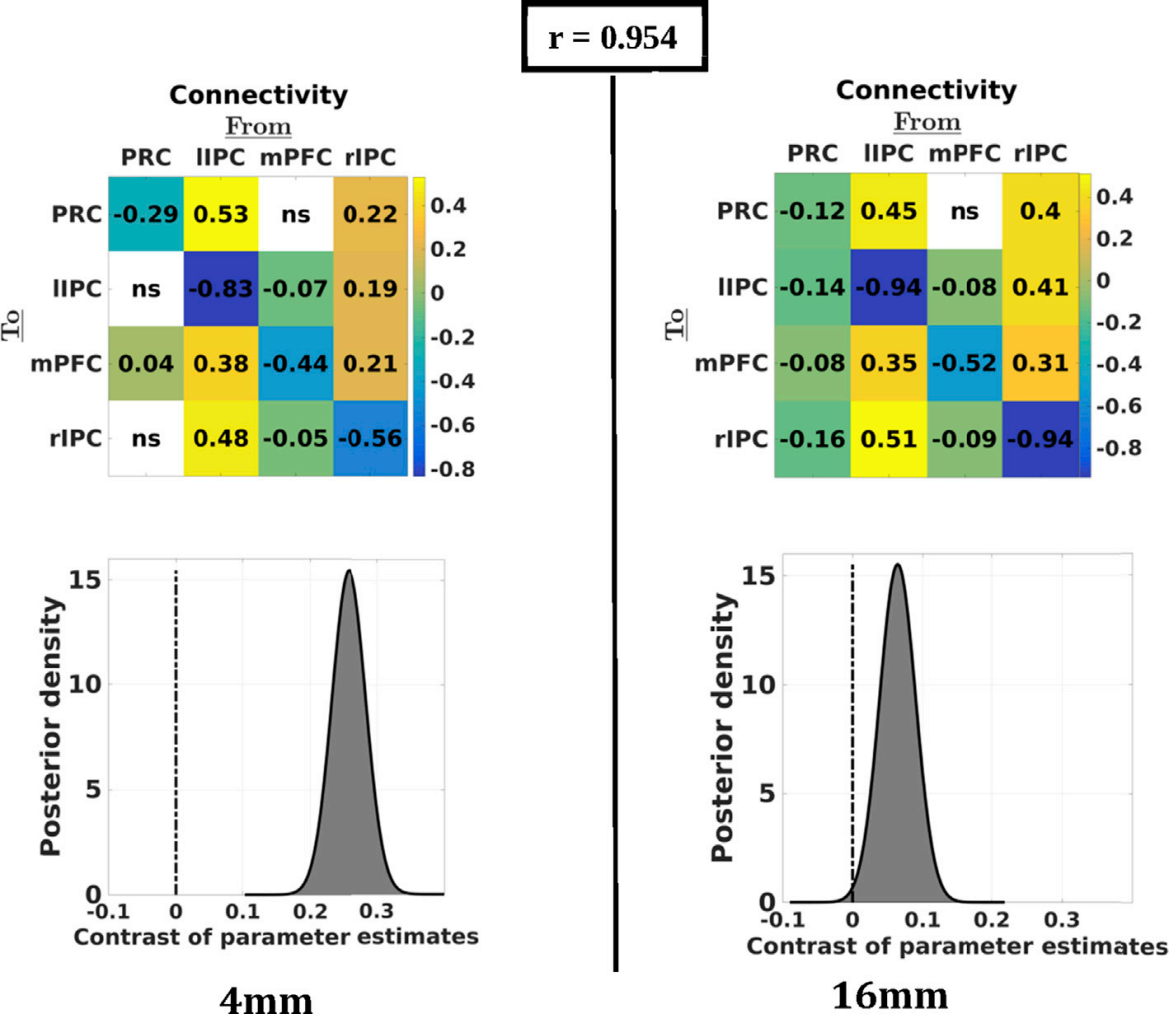
Group-average connectivity for the smallest and largest ROI sizes. Upper panels: effective connectivity matrices for each ROI size at the group-level. Upper number indicates the correlation between (vectorised) matrices. Lower panels: asymmetry of the group-level network (positive = left dominant; negative = right-dominant). Larger ROI sizes yielded less asymmetry at the group level. However, even at bigger ROIs the network was left-dominant (see posterior probability). *Ns* depicts not significant (i.e., posterior probability below 90%).

Next, we assessed the influence of ROI size on connectivity at the group- and subject-level, as well as its influence on reliability. Group-level results are shown in Fig. 6. Generally, connectivity patterns were very similar for different ROI sizes at the group-level (mean correlation = 0.980; range = 0.954–0.997). Asymmetry decreased with increasing ROI size (see Fig. 6, lower panel), but was left dominant, even for larger ROI sizes (posterior probability > 0.90). At the subject level (not shown), hemispheric asymmetry did not change for any ROI size in 11 subjects (65%), while in three subjects (18%) hemispheric asymmetry flipped for some ROI sizes. The *between-subject* similarity (i.e., average correlation between all pairs of subjects' connectivity matrices) was 0.59, 0.61, 0.56, and 0.64 for increasing ROI radii. The same statistical tests as above were used to test the difference in similarity between consecutive ROI sizes (i.e., 4 vs. 8 mm, 8 vs. 12 mm, 12 vs. 16 mm), using an FDR of 5% (N = 3). This procedure showed that the consistency of connectivity estimates did not change significantly between any ROI size (Effect size = [0.023 0.025 0.078]; uncorrected p-value = [0.58 0.69 0.06]; FDR-corrected p-value = [0.69  0.69 0.19], for the three contrasts, respectively). The reliability of connectivity between *session-specific* connectivity matrices, assessed as the average correlation between effective connectivity matrices, was 0.40, 0.39, 0.43, and 0.41 for increasing ROI radii (i.e., 4, 8, 12, and 16 mm), showing that the size of the ROI did not have a discernible influence on the reliability of connectivity estimates. The same tests for significance between consecutive ROI sizes were performed for each subject, which showed that only one subject's stability (subject 2) changed significantly between two ROI sizes (i.e., between 12 and 16 mm; Effect size = 0.02; uncorrected p-value = 0.001; FDR-corrected p-value = 0.036).

#### 3.4.3 Effect of global signal regression

**Fig. 7:**
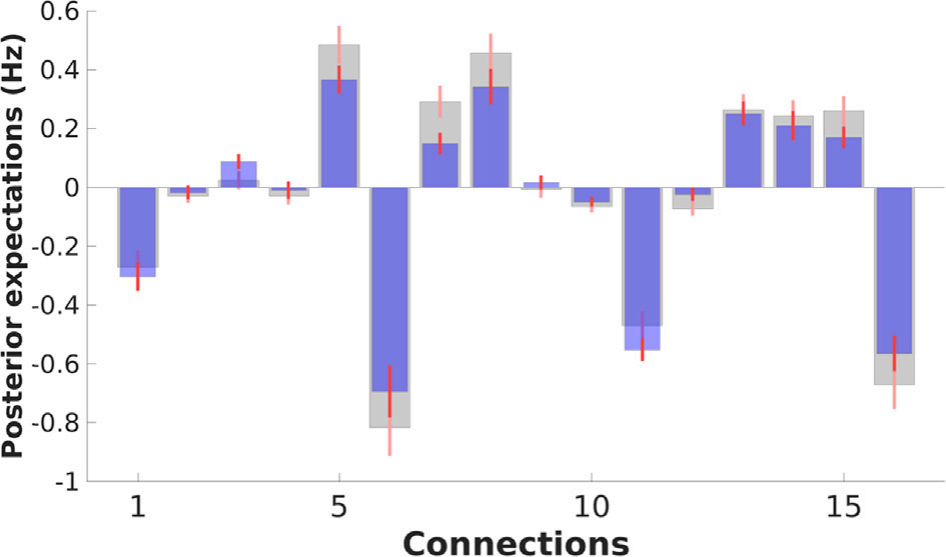
Group-average connectivity with GSR (blue bars), with narrow red bars showing 90% confidence intervals (i.e., Bayesian credible intervals) and without GSR (grey bars). Generally, extrinsic connectivity decreased in magnitude, while intrinsic connections changed in either positive or negative direction. However, no dramatic changes (e.g., significant changes in sign) were found.

Connectivity with and without GSR is shown in Fig. 7. At the group-level, extrinsic connectivity decreased slightly in magnitude after GSR (mean decrease = 0.05 Hz), while intrinsic connectivity changed in either a negative or positive direction (depending on the specific region). Importantly, no sign flips were observed. At the subject level, 14 (82%) subjects' asymmetry patterns were unaffected by GSR, although asymmetry was often less pronounced after GSR. The between-subject similarity of connectivity matrices did not change significantly after GSR (Effect size = 0.04; Number permutations = 50000; p = 0.076). The within-subject reliability of connectivity patterns was very similar after GSR (average within-subject correlation was 0.39 and 0.37 without and with GSR, respectively). The statistical significance of this difference was assessed for each subject using 10000 randomization and an FDR of 5% (N = 17). This procedure showed that one subject's (subject 2) reliability changed significantly after GSR (Effect size = −0.036; uncorrected p-value = 0.002; FDR-corrected p-value = 0.039).

## 4 Discussion

In this study, we combined fMRI data from multiple longitudinal studies to investigate within and between subject variability of effective connectivity. Collectively, spectral DCM furnished robust connectivity estimates for each subject, enabled us to track changes in fluctuations across scan sessions, and allowed us to draw conclusions beyond participant groups, scanners, and scanning parameters. Our results suggest that, across datasets, individuals consistently show hemispheric asymmetry in effective connectivity within the default mode network. Previous studies using spectral DCM have also shown some levels of asymmetry at the group-level. Razi et al. (2015) and Zhou et al. (2018) found larger influence from left compared to right parietal cortex, while Sharaev et al. (2016) found the opposite pattern (although the left-right difference was small). Moreover, all studies found that the parietal cortex has a driving influence in the core DMN (Zhou et al., 2018). The present study reproduced the latter result for individual subjects, and suggests that the (small) differences between studies might be attributed to a difference in lateralization patterns of the individual subjects studied. It is worth noting that we found that self-inhibition was lowest for the dominant IPC in 15 out of 16 asymmetric subjects. This makes sense from a network perspective; since a region that dominates the network should indeed show prolonged (i.e., disinhibited) activity. This observation suggests that the parameters estimated by spectral DCM covary in an intuitive and consistent way.

Lateralization of the default mode network has also been found in other studies using functional connectivity (e.g., Agcaoglu et al., 2015; Nielsen et al., 2013). Agcaoglu et al. (2015) applied independent component analysis to a large group of subjects (n = 603) and found that almost all default mode network components were left lateralized. Similarly, Nielsen et al. (2013) showed that many left-lateralized resting state hubs are part of the default mode network. Our results complement these studies, in showing that hemispheric asymmetry is expressed in terms of effective connectivity, and that the asymmetry is mainly present for interhemispheric connections and connections from lateral to medial areas. Moreover, we have shown that hemispheric asymmetry of effective connectivity is a source of between-subject variability explaining more than 50% of between-subject variance.

The pattern of hemispheric asymmetry was reliable for several subjects: Asymmetry was found to be stable (i.e., effect size > 70%) in twelve subjects and the null-hypothesis of random asymmetry could be rejected for 9 out of 16 asymmetric subjects. Furthermore, individual connections arising from either right or left IPC showed high sign-stability, which coincided with the individual's asymmetry. Longitudinal studies assessing the reliability of functional connectivity (e.g., Choe et al., 2015; Gordon et al., 2017) do not usually focus on hemispheric asymmetry. However, hemispheric specialization is an important issue in cognitive neuroscience (see, e.g., Hervé et al., 2013). Crucially, a change in DMN lateralization has been associated with psychiatric syndromes (see, e.g., Swanson et al., 2011). The overall stability of the asymmetry of effective connectivity in the DMN could speak to its use as a biomarker for future studies. We also found that some subjects showed more variable hemispheric asymmetry. Within-subject variations in connectivity patterns have also been observed in longitudinal functional connectivity studies. Gordon et al. (2017) found that one specific participant showed considerable lower reliability compared to the others, which they attributed to a higher level of drowsiness. Although this precise subject was left out of the present analyses, it is likely that subject or session specific characteristics (e.g., emotionality) might have caused more variable asymmetry in some subjects compared to others.

It is interesting to compare variability in asymmetry at the subject-level to variability at the session-level. Our results suggested that asymmetry plays a more prominent role for between-subject variability compared to between-session variability. First, only 8 out of 16 lateralized subjects showed (within-subject) asymmetric loadings on the first principal component. Second, the explained within-subject (i.e., over sessions) variance of the first principal component was lower in 7 out of 8 subjects (i.e., subjects having asymmetric PCA loadings) compared to the explained variance at the between-subject level. Third, the increase in within-subject similarity after swapping left-right labels was lower than the increase in between-subject similarity after doing similar swapping at the session-level (increase in similarity was 28% at the between-subject level vs. 13% at the between-session level). In fact, only one out of 17 subjects showed more increase in stability when swapping labels compared to the between-subjects case. Of course these findings do not provide conclusive evidence that asymmetry does not play a role at the within-subject level for some subjects. However, variability in asymmetry between sessions might be explained by the fact that the resting state has few constraints (i.e., the subject receives little instruction), and therefore asymmetry (or lateralization) is likely to change between sessions in some participants. Asymmetry might depend on many factors, including emotional state, amount of sleep, or physiological processes (e.g., caffeine intake). The finding that some participants showed more stability in asymmetry compared to other participants might also be related to subject-specific stability of emotional, behavioural, and/or physiological processes.

The correlation between session-specific effective connectivity matrices, reflecting reliability of whole network connectivity, was on average (across subjects) around 0.4 (range = 0.08–0.71). Studies assessing reliability of resting-state *functional* connectivity using longitudinal datasets (e.g., Gordon et al., 2017) have typically reported higher values of reliability of whole-brain connectivity matrices (average correlation > 0.60). The lower correlation found in the present study might be attributed to the fact that we focused on a specific network, while other studies focused on whole-brain connectivity. Possibly, network-specific reliability is lower compared to whole-brain reliability of connectivity. In light of this, Pannunzi et al. (2017) showed that whole-brain functional connectivity is more reliable than single connections. Furthermore, reliability of connectivity could depend on the specific network in question. Regarding identifiability, Finn et al. (2015) showed that some networks (namely the frontoparietal networks) contribute more to identifiability of individual subjects' connectivity matrices than other networks, which might be related to a difference in reliability.

To assess the effect of processing methods on connectivity and reliability, the analyses were repeated using global signal regression, varying ROI sizes, and (empirical) Bayesian model reduction. Generally, processing techniques had little effects on our results. Global signal regression had no effect on hemispheric asymmetry for most subjects (82% of subjects) and did not alter the (within-subject) stability of effective connectivity. Such robustness is quite remarkable, given that the global signal is an important subject of debate in many functional connectivity studies (see e.g., Murphy and Fox, 2017). The robustness of spectral DCM to global confounds is probably explained by the fact that global fluctuations in fMRI signal are modelled explicitly. In other words, parameters representing different sources of noise that are included in DCM can capture fluctuations in global signal that is not mediated by changes in effective connectivity.

Similarly, ROI size had no effect on asymmetry for most subjects (11 out of 17 subjects) and did not have an impact on the reliability of connectivity patterns. Importantly, Bayesian model reduction (BMR; Friston et al., 2016) increased both within- and between-subject consistency of effective connectivity patterns. This suggests that the use of subject and group-specific priors to update the parameters at the session and subject level may enhance reliability, by increasing the probability that parameters are drawn out of local extrema towards the subject or group mean.

Generally, the results raise the question of what might explain the differences between subjects (e.g., hemispheric asymmetry, connection types) and fluctuations within subjects. Variability between subjects might be explained by several factors. First, variability might be related to subject-specific characteristics such as age, gender, and intelligence level. Agcaoglu et al. (2015), for example, showed that age and gender are related to a difference in asymmetry in some resting state networks, most notably the visual network. Similarly, Joliot et al. (2016) showed a relationship between language lateralization and lateralization of resting state connectivity. Our sample comprised participants with a wide age-range (between 24 and 45 years) and was balanced with respect to gender (55% females). Possibly these subjects' characteristics might have played a role in the extent of asymmetry or at the level of individual connections. Second, differences in scan procedures and sequences might explain observed differences between subjects. Resting state scans are acquired when subjects have either their eyes open or closed; however, no consistent paradigm has been adopted. Studies have shown that a difference in ‘eyes open’ versus ‘eyes closed’ conditions might have an impact on connectivity and reliability during rest (e.g., Zhang et al., 2015; Zou et al., 2015). In our study, the ‘day2day’ dataset was acquired under ‘eyes closed’ conditions and showed a higher proportion of left dominant subjects compared to the ‘MSC’ dataset, which was acquired during ‘eyes open’ conditions. Such procedural differences might thus explain observed differences among subjects. Although several accounts can be offered for between-subject differences, our sample size was too small (n = 17 after exclusion) to support robust explanations. Future studies using datasets with more subjects could try to address possible sources of between-subject variability in effective connectivity, while accounting for within-subject variability.

Fluctuations within individuals might also be explained by several factors. First, fluctuations in effective connectivity might be related to day-to-day changes in mood, behaviour (e.g., amount of sleep), or physiology (e.g., hormonal cycle). Some studies have shown that functional connectivity within the DMN is related to sleepiness (Ward et al., 2013) and is diminished after sleep deprivation (De Havas et al., 2012). Other studies found an influence of female hormones on functional connectivity during rest (e.g., Pletzer et al., 2016). Second, between-subject fluctuations in connectivity might be explained by differences in regional or global noise. Although we did not find a notable difference in parameter estimates with and without GSR, this is not explicit evidence for an influence of global signal on connectivity (or its absence). Similarly, if region-specific noise levels change across sessions, parameter estimates might be affected by conditional dependencies between connectivity and (scale free) noise estimates. Third, Park et al. (2017) have shown that effective connectivity during resting state fluctuates on a short time scale. These faster fluctuations might cause differences among scanning sessions, and therefore explain longitudinal variability in effective connectivity. Indeed, Park et al. (2017) showed that between-session consistency increased when within-session fluctuations were taken into account.

Longitudinal datasets afford the opportunity to test the above hypotheses. Such analyses however fall outside the scope of the present study. Our aim was to provide a framework to test both between- and within-subject variability and consistency of effective connectivity in a single design. The use of PEB, upon which this framework was build, allows researchers to assess relations between connectivity patterns (e.g., asymmetry) and other measures (e.g., physiology), which is of great importance for neuroscience. Future studies could address more refined accounts of between and within subject variability in further detail.

## Software availability

Code including all analyses steps and reproduction of results and figures is available at: https://github.com/halmgren/Pipeline_preprint_variability_reliability_DCM_rsfMRI.
