# Supplementary material for: Variability and reliability of effective connectivity within the core default mode network: A multi-site longitudinal spectral DCM study
Source: Neuroimage. 2018 Dec;183:757–68. doi: 10.1016/j.neuroimage.2018.08.053 (PMC6215332; doi:10.1016/j.neuroimage.2018.08.053)
Supplement: Multimedia component 1 [file mmc1.docx]

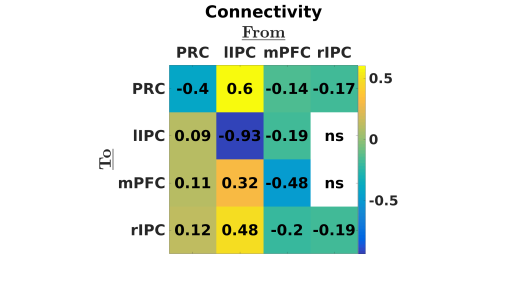

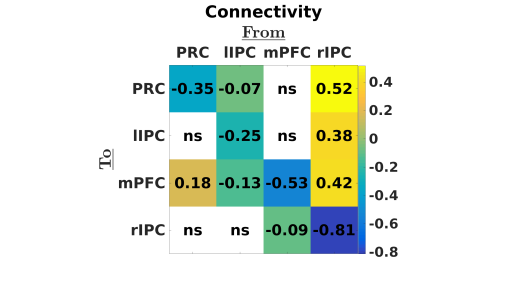

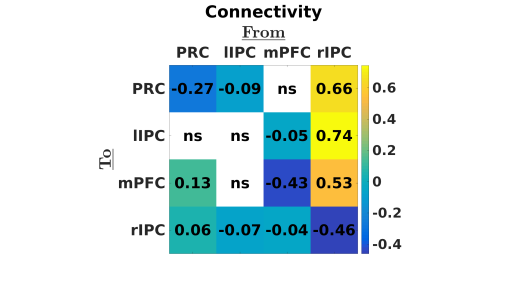

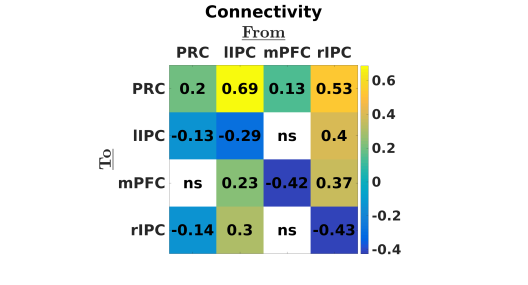

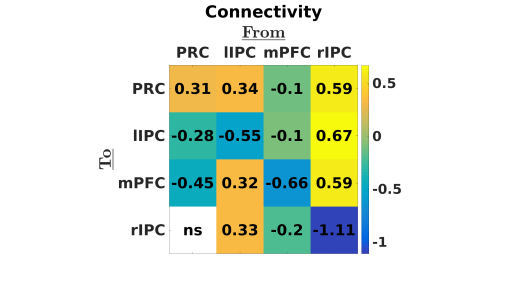

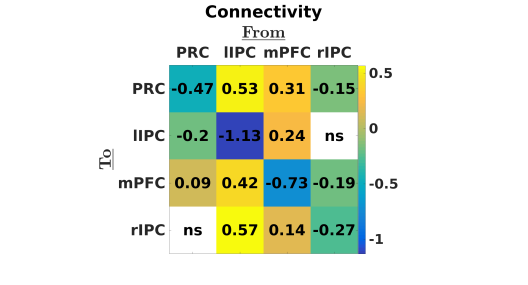

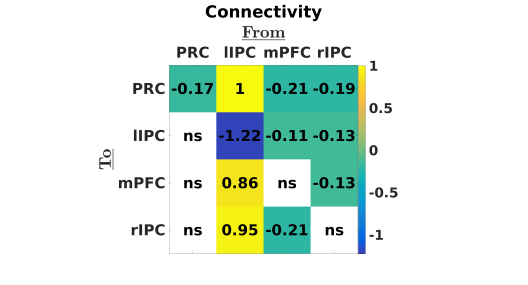

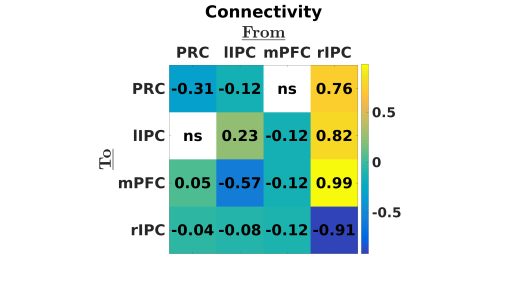

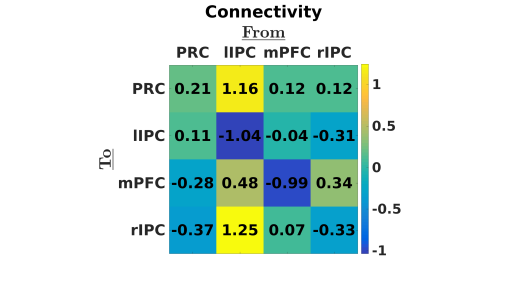

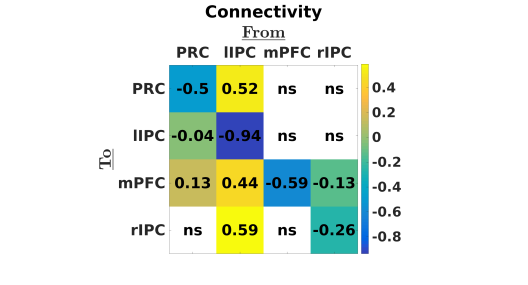

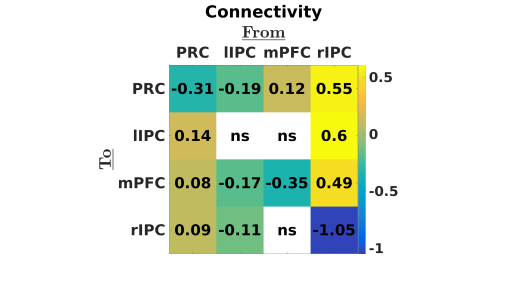

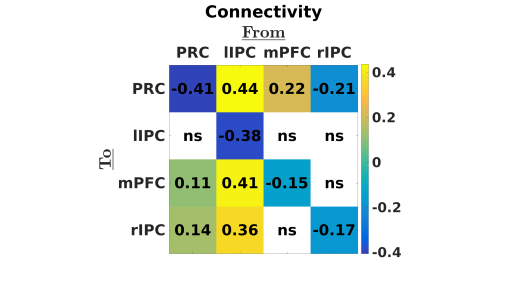

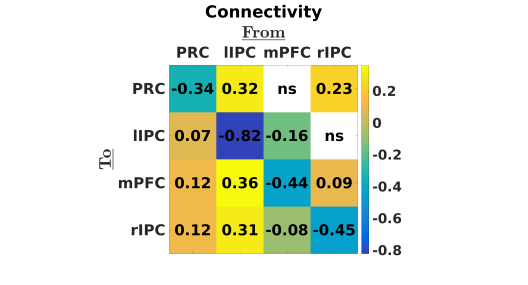

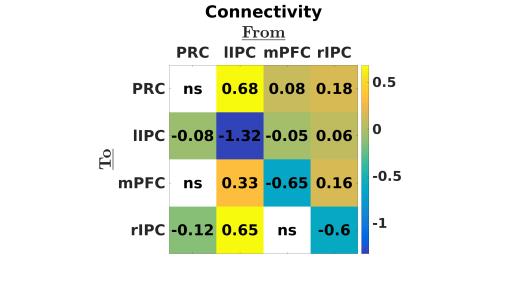

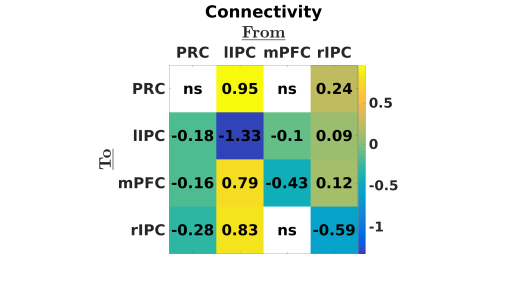

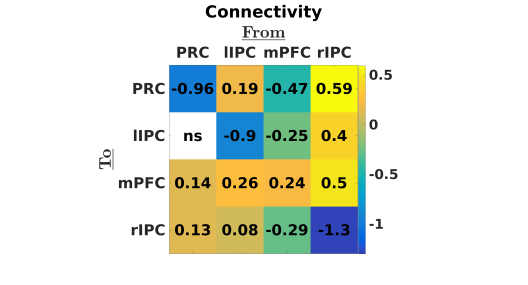

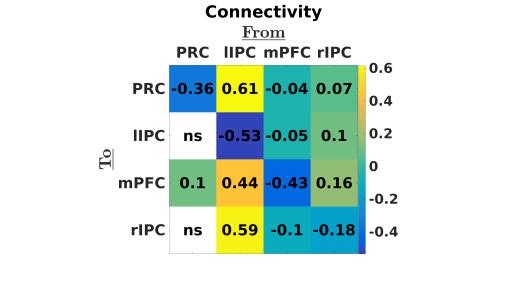


**S16**

**S11**

*Supplementary Figure 1.* Connectivity matrices for all subjects. Connections that did not reach the predefined posterior probability criterion of 90% were considered as non-significant (ns). All subjects, except subject 15, showed significant hemispheric asymmetry.

**S1**

**S20**

**S17**

**S15**

**S14**

**S12**

**S10**

**S9**

**S8**

**S6**

**S7**

**S5**

**S4**

**S3**

**S2**


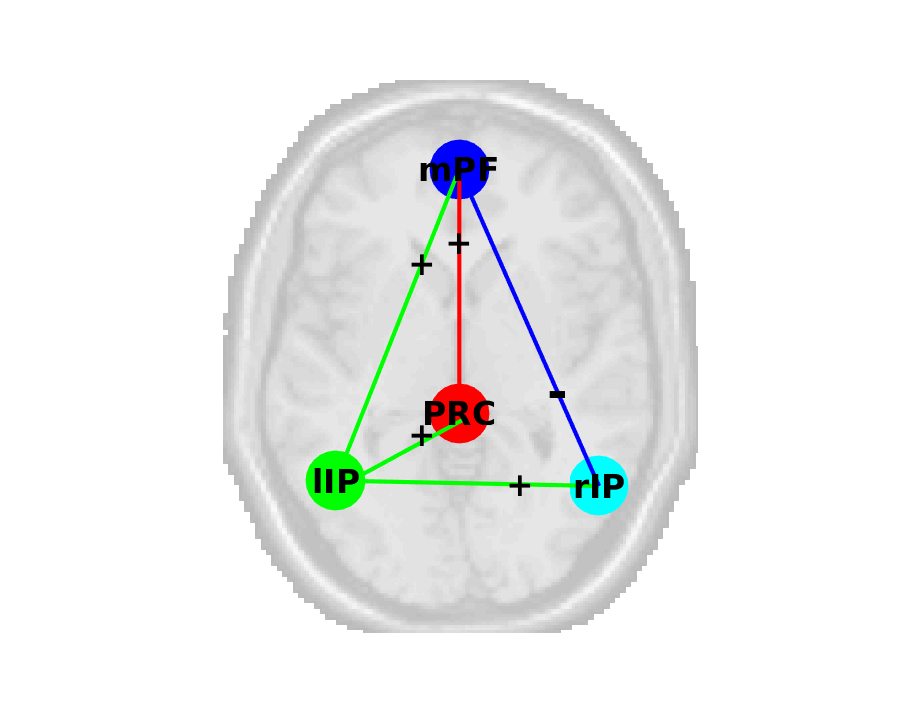

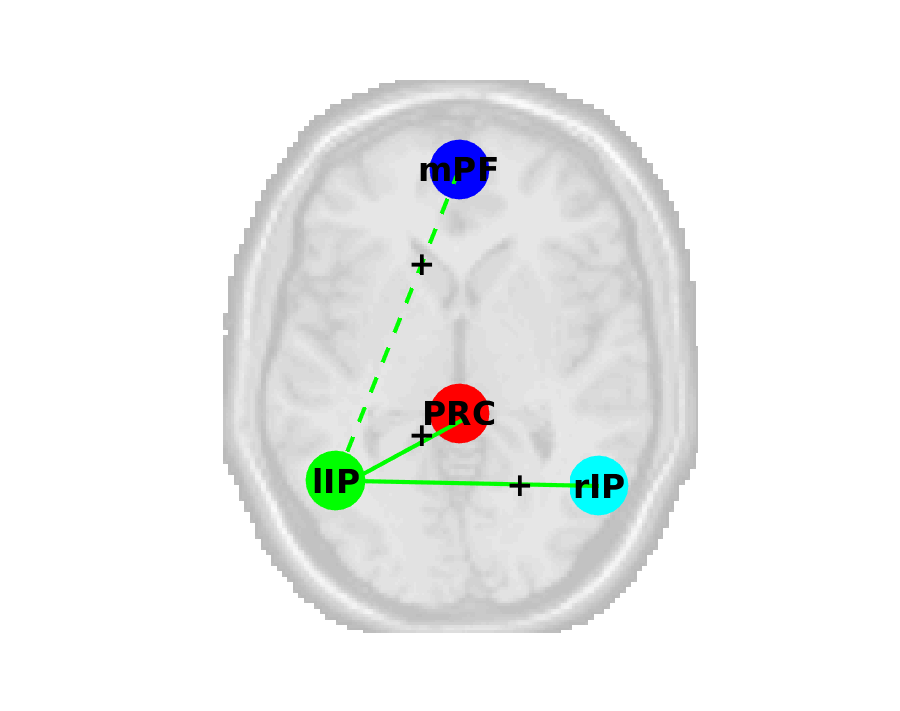

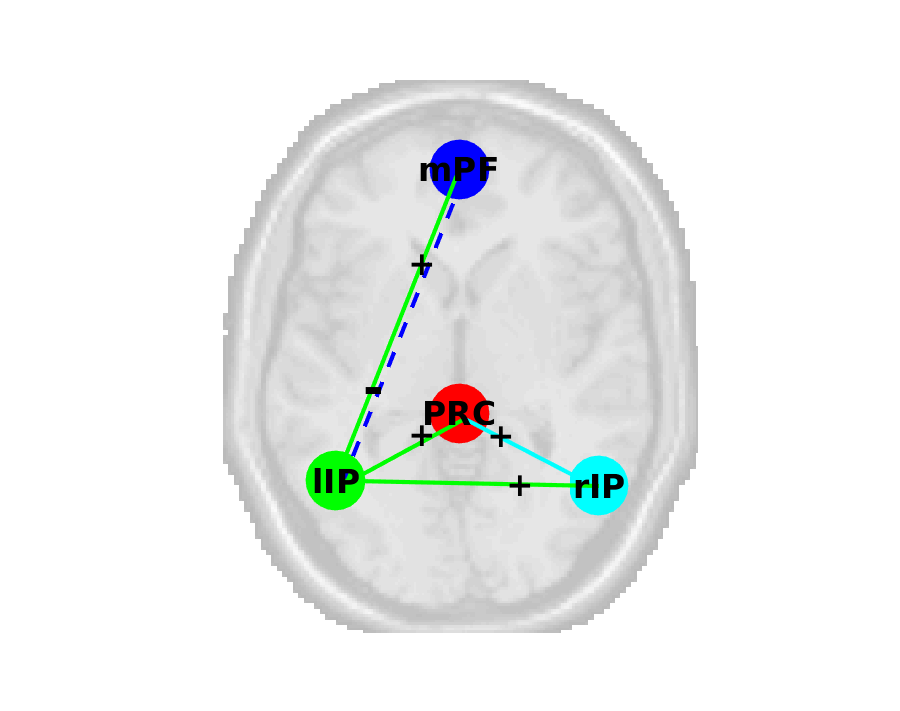

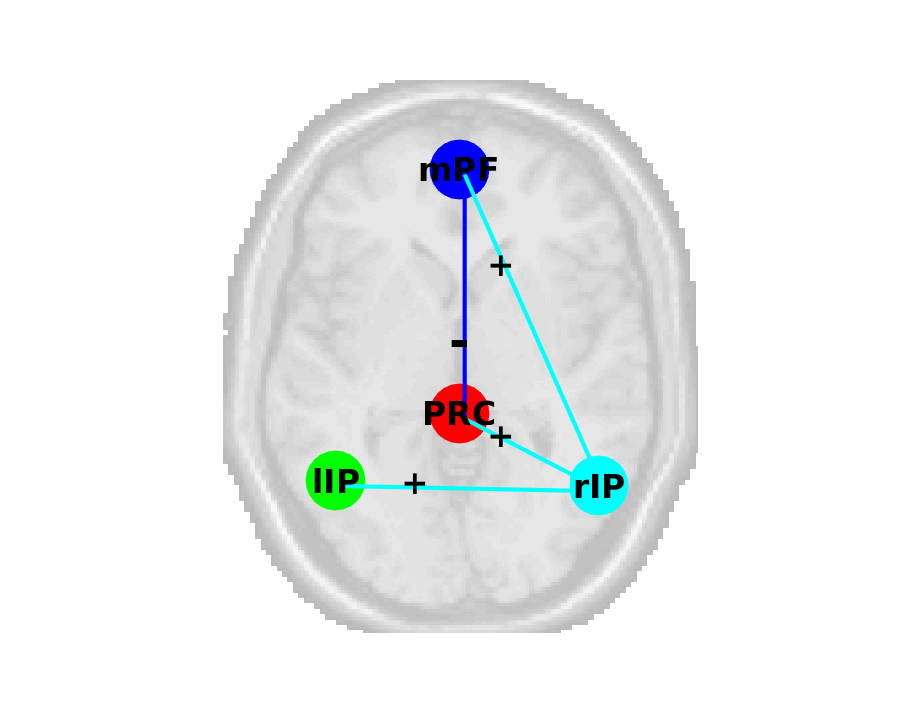

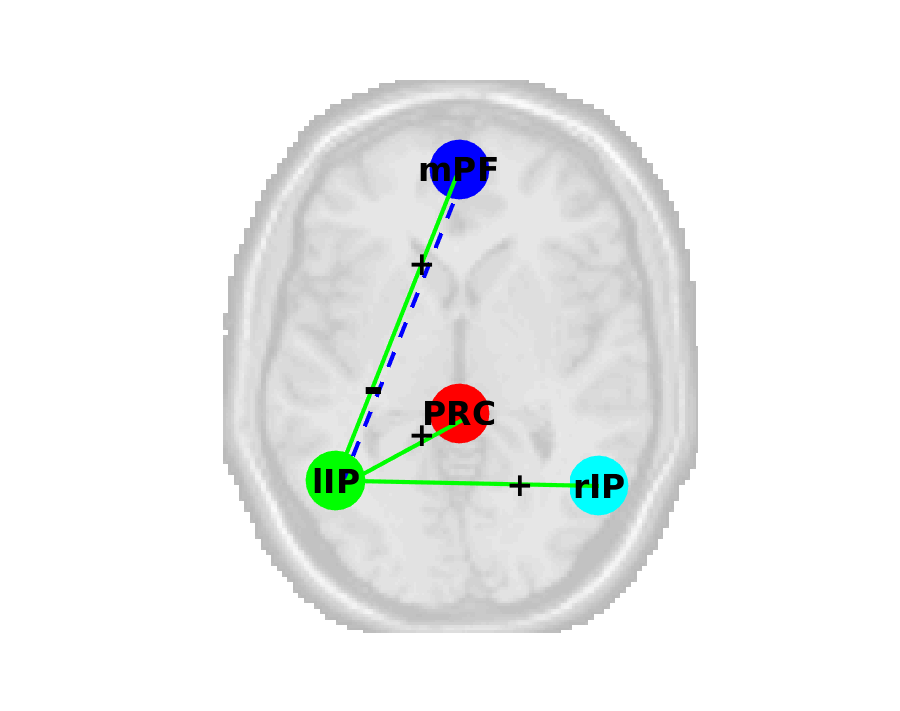

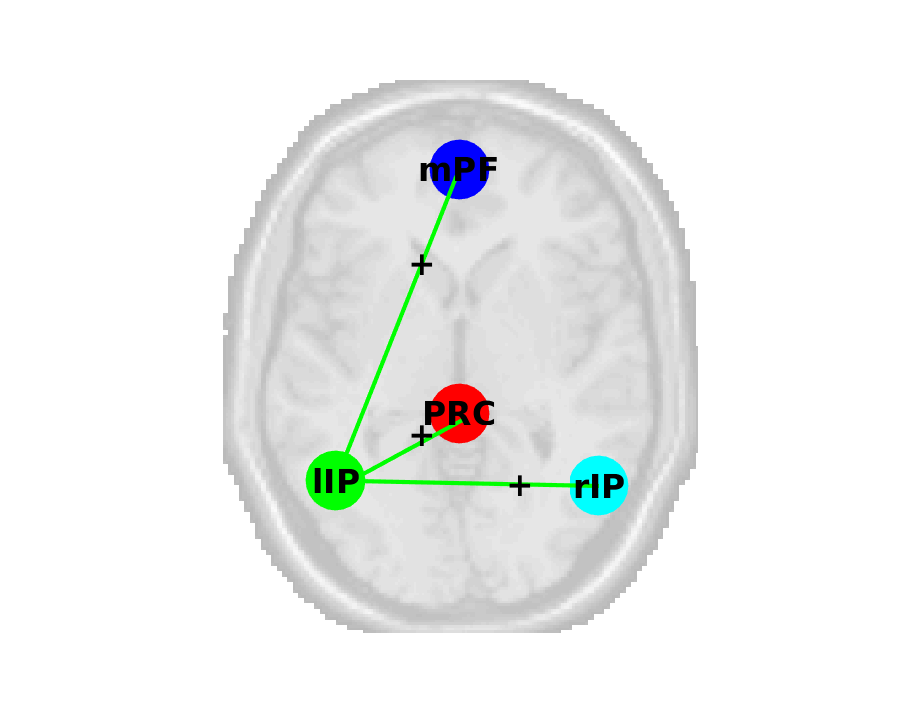

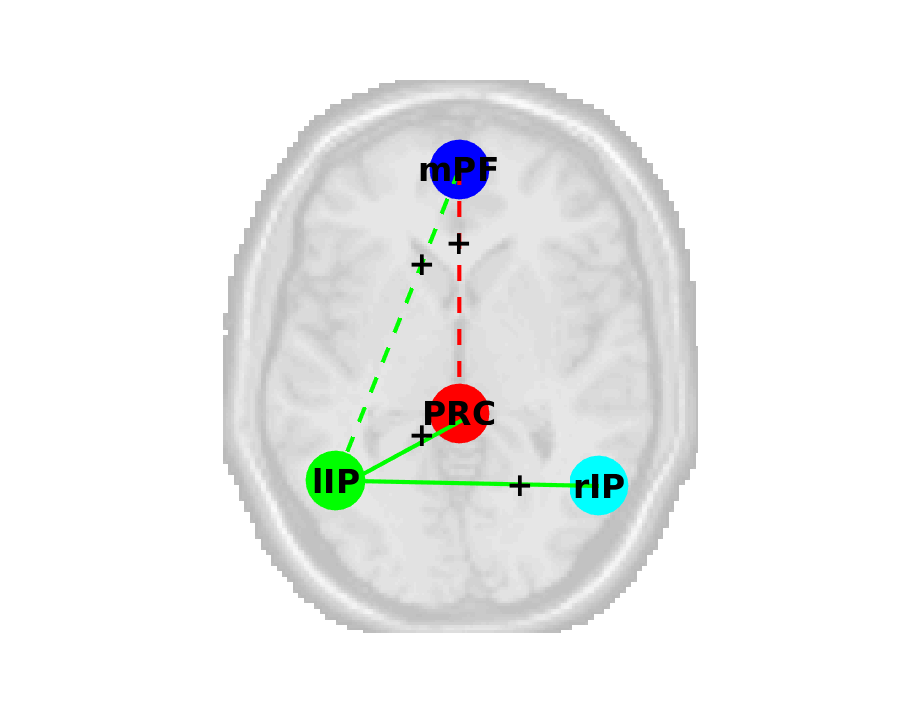

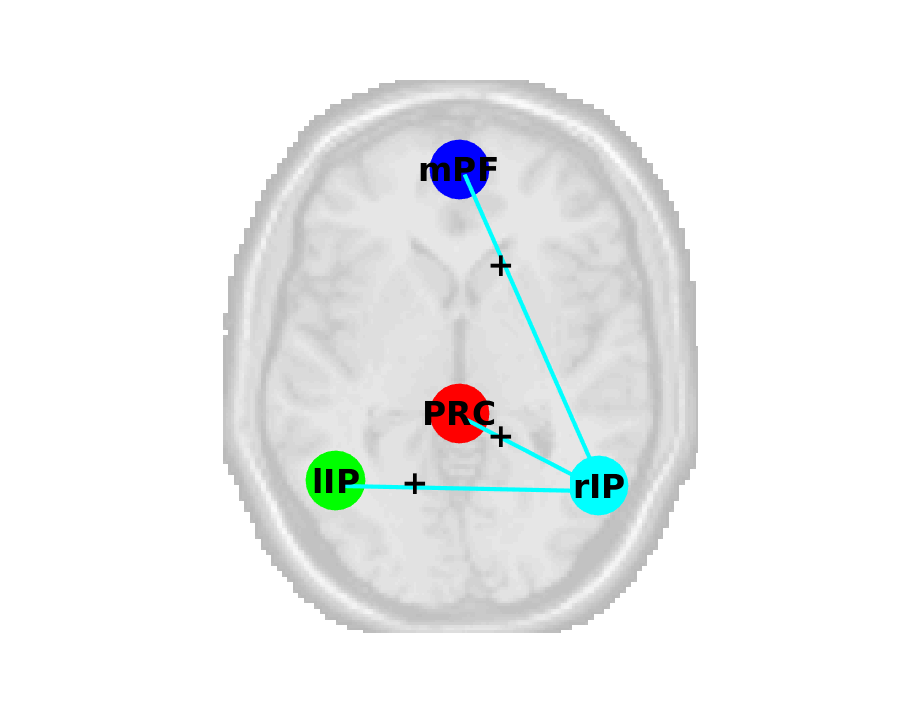

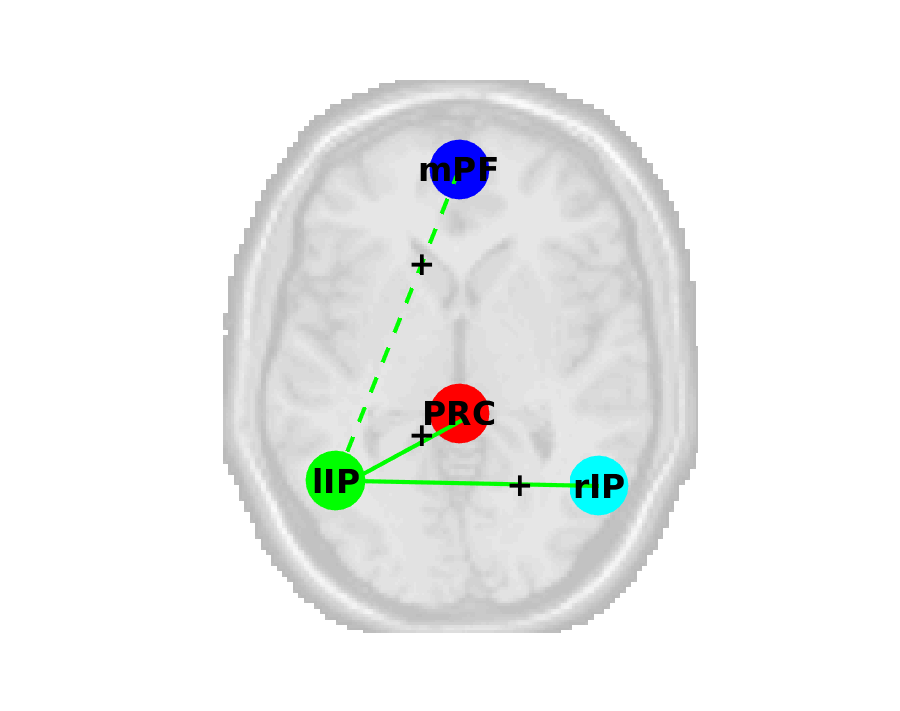

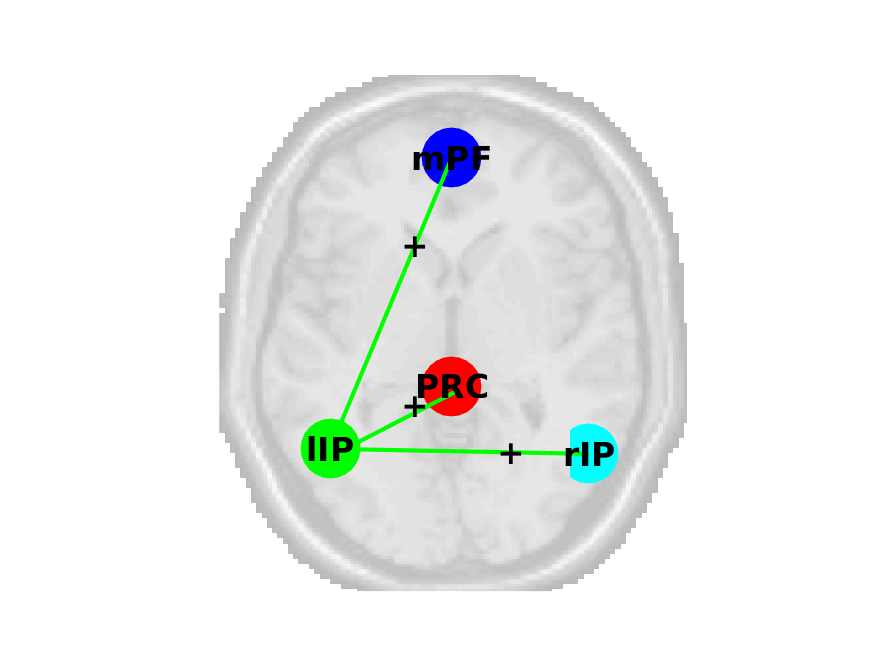

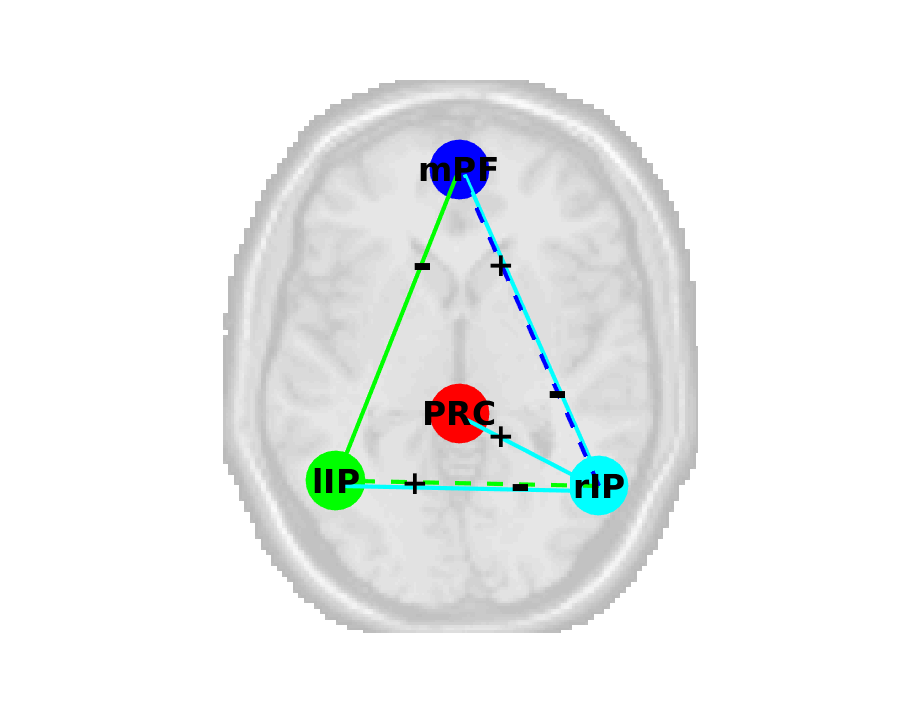

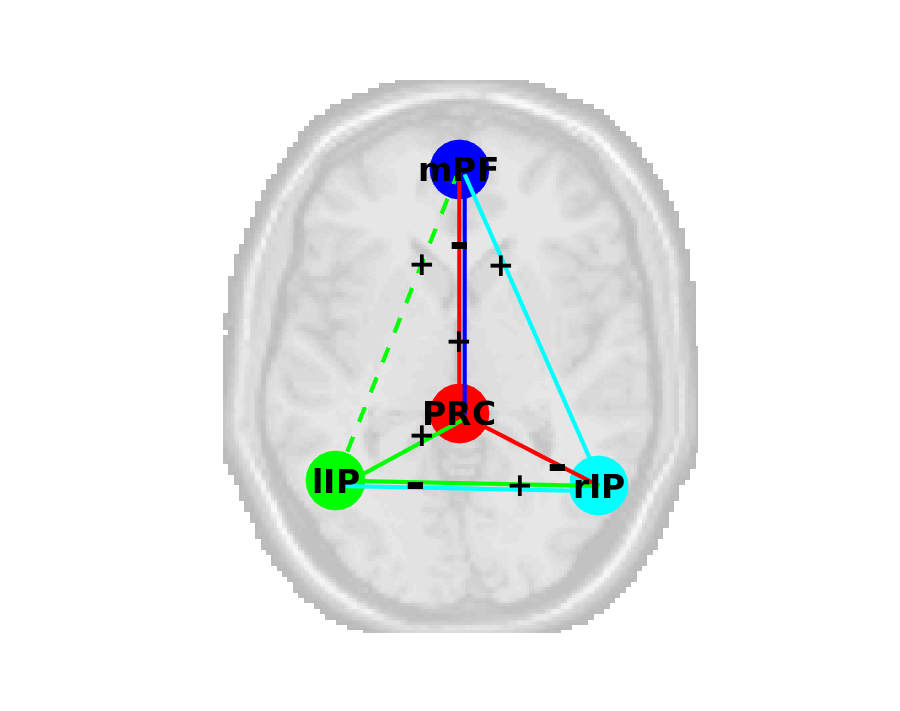

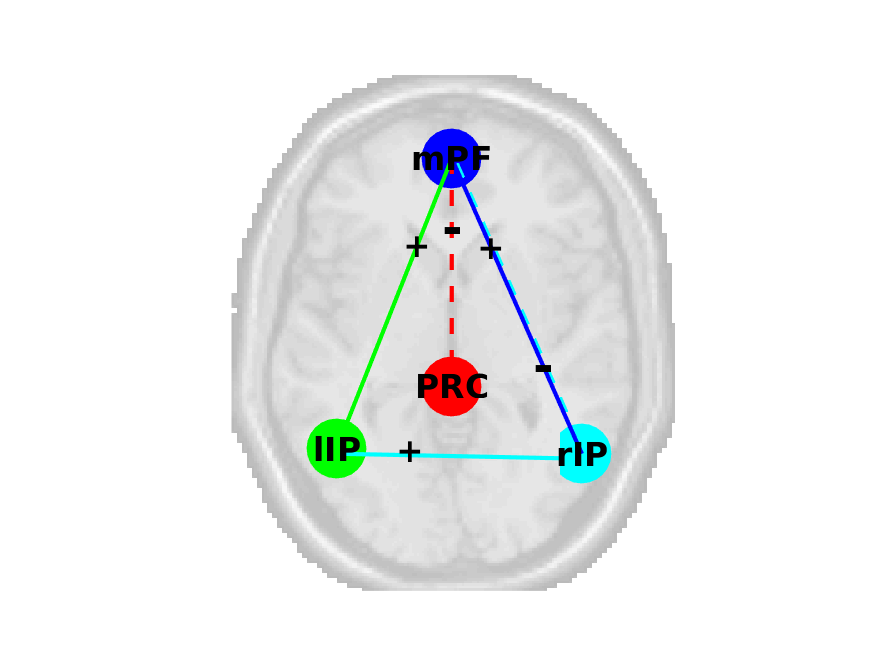

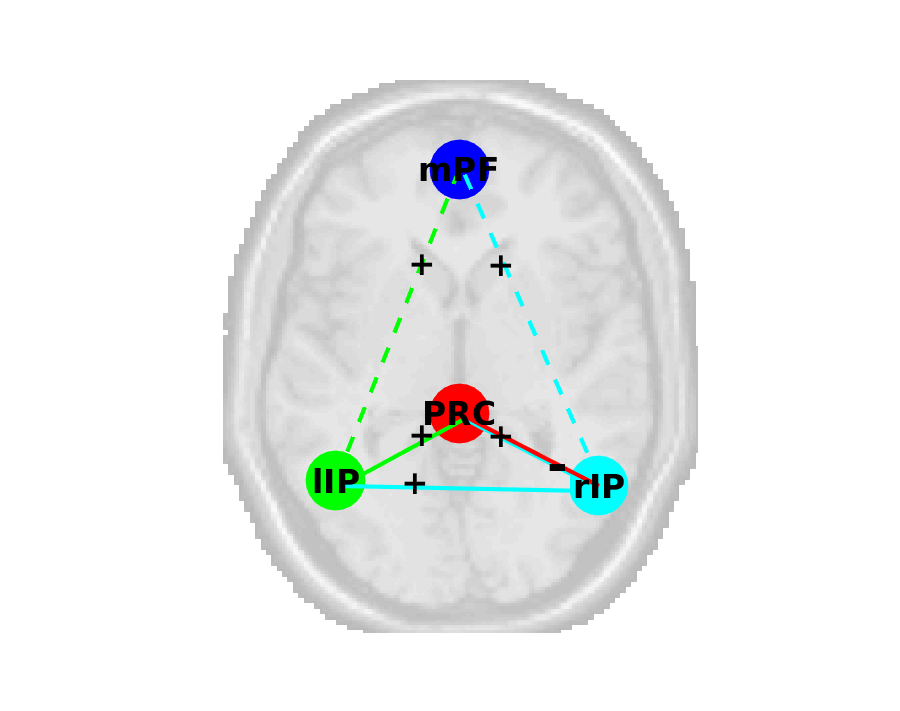

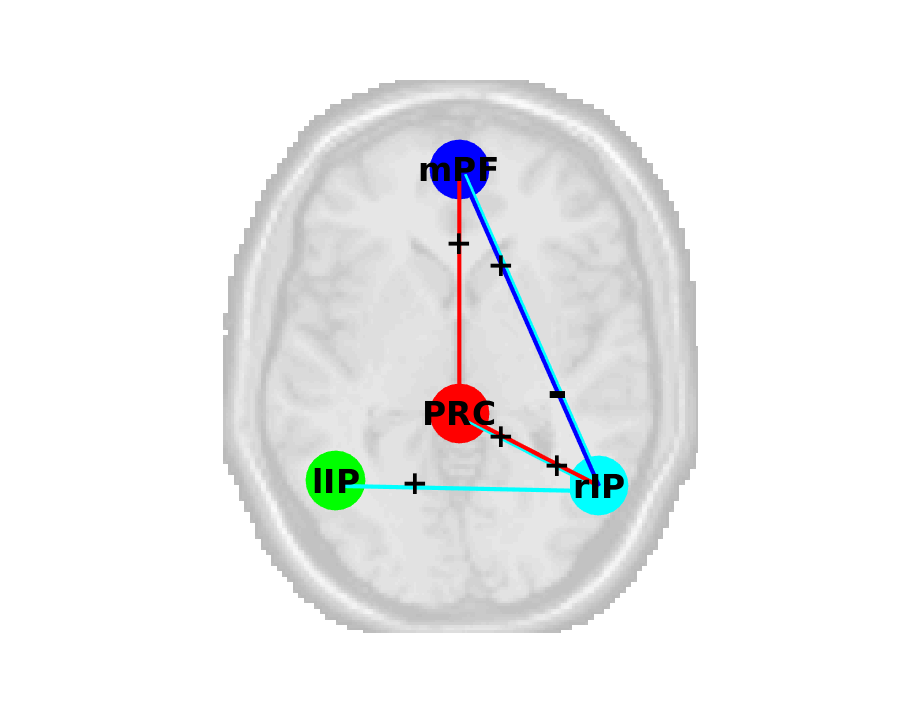

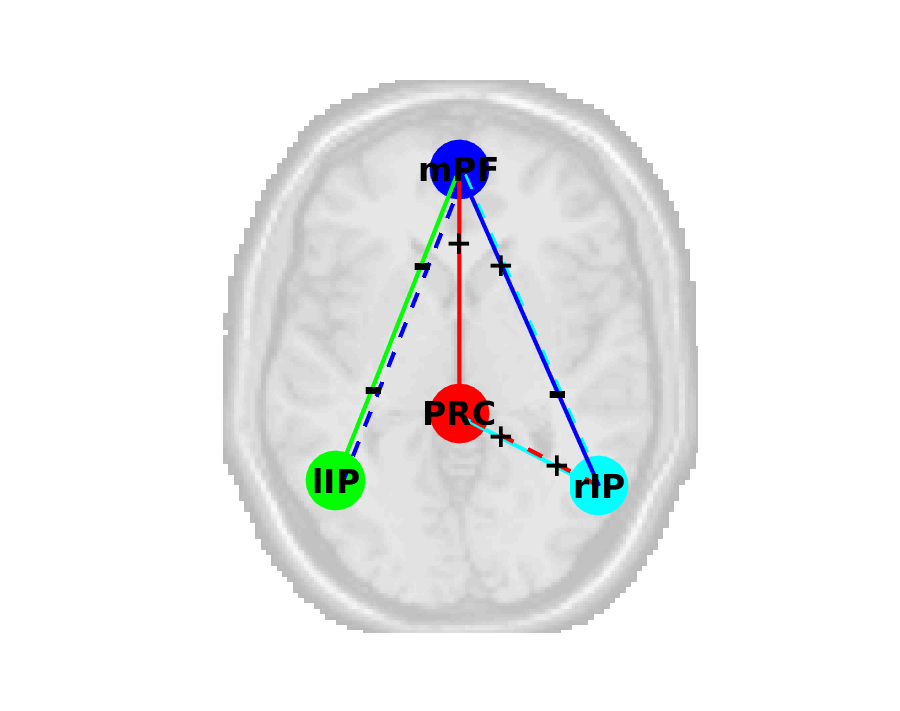

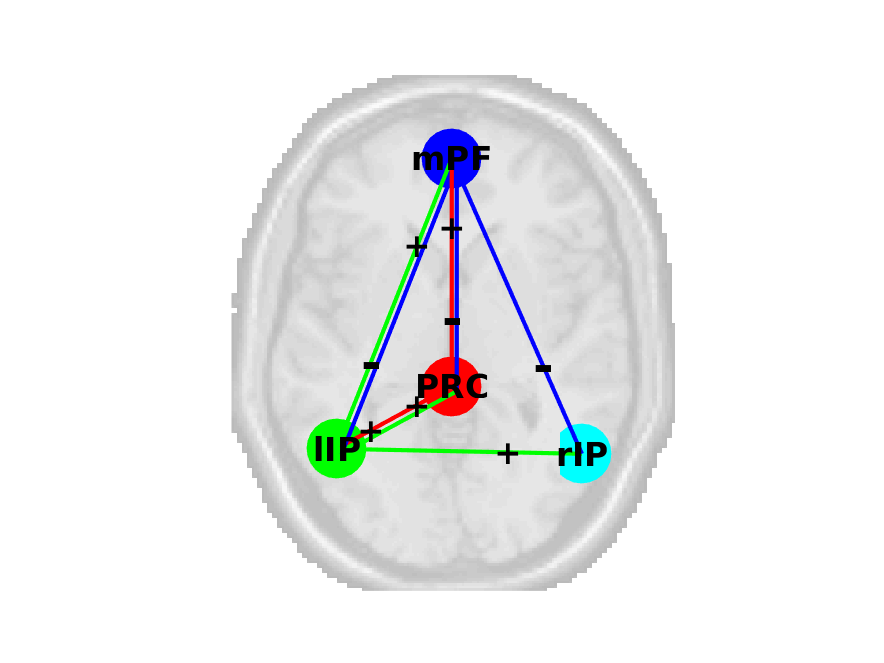


*Supplementary Figure 2.* Subject-specific sign stability. Connections that had the same time for 75% of sessions for the respective subject are shown using solid lines, connections that showed the same sign 70% of sessions are depicted with dashed lines. The line colours depict the source of a connection (e.g., green lines depict connections from the lIPC to other regions). Stable connections arise from left or right IPC for the majority of subjects, which coincides with the subject-specific asymmetry. For visualization purposes the precuneus is shown more anteriorly than in reality.

**S16**

**S15**

**S12**

**S10**

**S11**

**S7**

**S6**

**S3**

**S2**

**S20**

**S17**

**S14**

**S9**

**S1**

**S4**

**S8**

**S5**

| **Sub**  **Con** | **S1** | **S2** | **S3** | **S4** | **S5** | **S6** | **S7** | **S8** | **S9** | **S10** | **S11** | **S12** | **S14** | **S15** | **S16** | **S17** | **S20** |
| --- | --- | --- | --- | --- | --- | --- | --- | --- | --- | --- | --- | --- | --- | --- | --- | --- | --- |
| **p -> p** | **-0.13** | -0.31 | 0.33 | 0.11 | **-0.12** | -0.12 | **0.27** | **-0.06** | **-0.31** | 0.27 | **0.07** | 0.46 | 0.21 | **0.22** | **0.01** | 0.51 | 0.21 |
| **p-> l** | **-0.01** | 0.35 | -0.28 | 0.08 | **0.16** | -0.14 | **-0.13** | **0.13** | **0.20** | -0.02 | **-0.01** | -0.40 | -0.17 | **-0.01** | **0.04** | -0.42 | -0.08 |
| **p -> m** | **0.07** | 0.30 | -0.17 | -0.18 | **0.12** | 0.01 | **-0.00** | **0.13** | **0.11** | 0.06 | **-0.01** | -0.08 | -0.02 | **-0.01** | **0.01** | -0.25 | 0.04 |
| **p -> r** | **0.08** | 0.42 | -0.51 | -0.37 | **0.18** | 0.10 | **-0.04** | **-0.08** | **0.06** | -0.15 | **-0.07** | -0.07 | -0.39 | **-0.03** | **0.02** | -0.18 | -0.27 |
| **l -> p** | **0.42** | -0.09 | 0.06 | 0.12 | **0.33** | 0.01 | **0.46** | **0.29** | **0.55** | -0.22 | **0.27** | -0.13 | 0.47 | **0.37** | **-0.30** | 0.13 | -0.37 |
| **l -> l** | **-0.29** | 0.28 | -0.21 | -0.04 | **-0.08** | -0.09 | **-0.27** | **-0.24** | **-0.17** | 0.03 | **-0.46** | 0.09 | -0.32 | **-0.09** | **0.60** | -0.07 | 0.24 |
| **l -> m** | **0.21** | -0.09 | 0.04 | 0.40 | **0.10** | -0.34 | **0.46** | **0.18** | **0.36** | -0.16 | **0.10** | 0.02 | 0.13 | **0.22** | **-0.26** | 0.03 | -0.07 |
| **l -> r** | **0.28** | -0.21 | 0.51 | 0.35 | **0.31** | -0.26 | **0.21** | **0.51** | **0.20** | 0.40 | **0.40** | -0.02 | 0.60 | **0.38** | **-0.25** | -0.06 | 0.30 |
| **m -> p** | **-0.05** | -0.10 | 0.07 | -0.27 | **-0.02** | 0.51 | **0.08** | **-0.13** | **-0.20** | -0.22 | **0.09** | -0.02 | -0.03 | **0.07** | **-0.01** | 0.07 | -0.15 |
| **m -> l** | **-0.04** | -0.10 | 0.09 | -0.26 | **0.01** | 0.34 | **-0.00** | **-0.08** | **-0.22** | -0.17 | **-0.02** | -0.06 | -0.00 | **0.02** | **0.02** | 0.09 | -0.09 |
| **m -> m** | **0.10** | 0.35 | -0.21 | 0.17 | **0.18** | -0.39 | **0.12** | **0.09** | **0.03** | 0.48 | **-0.15** | 0.14 | 0.05 | **0.04** | **0.08** | -0.05 | 0.29 |
| **m -> r** | **0.02** | -0.17 | 0.11 | 0.12 | **-0.06** | 0.19 | **0.09** | **-0.08** | **0.02** | -0.26 | **0.05** | -0.11 | -0.03 | **0.08** | **-0.01** | -0.00 | -0.11 |
| **r -> p** | **-0.40** | -0.21 | 0.23 | 0.54 | **-0.44** | -0.39 | **-0.36** | **-0.23** | **-0.31** | 0.40 | **-0.24** | 0.44 | -0.21 | **-0.19** | **0.38** | 0.15 | 0.58 |
| **r -> l** | **-0.26** | -0.24 | 0.12 | 0.18 | **-0.37** | -0.20 | **-0.1** | **-0.39** | **-0.22** | 0.10 | **-0.39** | 0.55 | -0.15 | **-0.4** | **0.40** | 0.45 | 0.30 |
| **r -> m** | **-0.27** | -0.15 | 0.04 | 0.08 | **-0.22** | -0.10 | **-0.37** | **-0.23** | **-0.23** | 0.33 | **-0.15** | 0.19 | -0.06 | **-0.22** | **0.24** | 0.25 | 0.13 |
| **r -> r** | **0.52** | 0.29 | -0.28 | 0.02 | **0.52** | -0.03 | **0.25** | **0.47** | **0.24** | -0.02 | **0.52** | -0.14 | 0.01 | **0.60** | **-0.22** | -0.38 | -0.05 |
| **Expl.**  **Var.** | 36.7% | 26.3% | 34.3% | 35.7% | 24.5% | 41.8% | 34.0% | 24.0% | 25.5% | 26.2% | 59.1% | 49.6% | 53.7% | 41.4% | 72.8% | 45.3% | 58.6% |

*Supplementary Table 1.* Loadings on first principal component and respective proportion explained variance shown for all subjects. Loadings shown in bold have opposite signs for left and right inferior parietal cortex. Abbreviations: Sub/S = subject, Con = connection, Expl. Var = proportion explained variance. Anatomical labels: p = precuneus, l/r = left/right inferior parietal cortex, m = medial prefrontal cortex.
